# Priority Health Conditions and Global Life Expectancy Disparities

**DOI:** 10.1001/jamanetworkopen.2025.12198

**Published:** 2025-05-23

**Authors:** Omar Karlsson, Angela Y. Chang, Ole F. Norheim, Wenhui Mao, Sarah Bolongaita, Dean T. Jamison

**Affiliations:** 1 Centre for Economic Demography, School of Economics and Management, Lund University, Lund, Sweden; 2 Department of Economic History, School of Economics and Management, Lund University, Lund, Sweden; 3 Duke University Population Research Institute, Duke University, Durham, North Carolina; 4 Danish Institute for Advanced Study, University of Southern Denmark, Copenhagen, Denmark; 5 Danish Centre for Health Economics, University of Southern Denmark, Campusvej 55, 5230 Odense, Denmark; 6 Department of Global Health and Population, Harvard T.H. Chan School of Public Health, Boston, Massachusetts; 7 Bergen Centre for Ethics and Priority Setting (BCEPS), Department of Global Public Health and Primary Care, University of Bergen, Bergen, Norway; 8 Centre for Policy Impact in Global Health, Duke University, Durham, North Carolina; 9 Duke Global Health Institute, Duke University, Durham, North Carolina; 10 Institute for Global Health Sciences, University of California, San Francisco; 11 Department of Epidemiology and Biostatistics, University of California, San Francisco

## Abstract

**Importance:**

Life expectancy is a composite health measure reflecting acute and life-course exposures. Identifying conditions behind disparities in life expectancy can guide policy, planning, and financing to battle the most urgent health problems.

**Objective:**

To examine the contribution of 33 causes of death to life expectancy disparities, highlighting 2 sets of priority conditions—8 infectious and maternal and child health conditions (I-8) and 7 noncommunicable diseases and injuries (NCD-7).

**Design, Setting, and Participants:**

This cross-sectional study examined life expectancy disparities in 7 global regions and 165 countries from 2000 to 2021. Western Europe and Canada (hereafter referred to as the North Atlantic) in 2019 were used as a benchmark for life expectancy achievable with advanced health care and living standards. Life expectancy gaps in locations with life expectancy lower than the benchmark were decomposed by cause of death using the Pollard decomposition on the Global Health Estimates from the World Health Organization. Data were analyzed from February to March 2025.

**Exposure:**

Geographic location (countries and regions).

**Main Outcome and Measure:**

Life expectancy at birth.

**Results:**

In the median country in 2019, the I-8 and NCD-7 together accounted for 80% (IQR, 71%-88%) of the life expectancy gap compared with the North Atlantic. Outside sub-Saharan Africa, the NCD-7 accounted for the largest share of the gap; for example, more than the total life expectancy gap in China, or 5.5 (95% uncertainty bounds [UB], 5.0-6.0) years of a 4.3-year life expectancy gap; and 6.4 (95% UB, 5.9-6.8) years of a 11.5-year gap in India. However, reduced mortality from the I-8 contributed to enormous improvements in sub-Saharan Africa, accounting for 21.4 (95% UB, 20.6-22.2) years of a 31-year gap in 2000 and 11.4 (95% UB, 10.9-11.8) years of a 22-year gap in 2019. India transitioned from having most of the gap accounted for by the I-8 in 2000, or 11.9 (95% UB, 11.0-13.0) years of a 19.6-year life expectancy gap, to having a larger share accounted for by the NCD-7 in 2019.

**Conclusions and Relevance:**

This cross-sectional study suggests that a limited number of causes account for most life expectancy disparities. Together with current information on risk factors, interventions, and morbidity not yet reflected in life expectancy, the varying contributions of these causes to gaps in life expectancy can help focus health policy and guide interventions to reduce risk factors and treat conditions.

## Key Points

**Question:**

To what extent do 15 priority conditions contribute to life expectancy disparities across global regions and countries?

**Findings:**

This cross-sectional study suggests that in most cases, over 80% of life expectancy disparities were accounted for by the 15 priority conditions. Other causes of disparities studied—despite being broad—were not as prominent except in specific contexts.

**Meaning:**

These findings suggest limited resources should be directed toward high-impact areas, prioritizing interventions, controlling risk factors, and providing medical interventions for the conditions that contribute the most to life expectancy disparities.

## Teaser

This cross-sectional study evaluates the contributions of 33 causes of death to life expectancy disparities across global regions and countries.

## Introduction

Advances in public health and medicine, together with rising living standards, have greatly improved health as reflected, for example, in gains in life expectancy over the past 2 centuries.^1,2,3^ However, large health disparities suggest highly uneven improvements across countries.^4^ For example, while life expectancy at birth is 82 years in Western Europe, it is 62 years in sub-Saharan Africa.^5^ Limited capacity to finance and mobilize resources leaves full coverage of vital services out of reach for many countries.^6^ A focus on a limited number of highly cost-effective interventions targeted at conditions with a large or rising impact on health is more feasible.^7,8,9,10,11^

Here, we quantified how much 33 causes of death contributed to life expectancy gaps across global regions and countries. While the burden from different causes of death has been reported in various forms, this study anchors comparisons with a benchmark for life expectancy that has been demonstrated to be widely achievable with high living standards and advanced health care—that is, in Western Europe and Canada (hereafter referred to as the North Atlantic). Furthermore, we quantify the contributions of 2 sets of causes which are suggested priority conditions by the third *Lancet* Commission on Investing in Health^11^: (1) 8 infectious and maternal and child health conditions (collectively referred to as the I-8) and (2) 7 noncommunicable diseases and injuries (collectively referred to as the NCD-7).

Life expectancy serves as a reliable summary health indicator that reflects the composite impact of most adverse health exposures and morbidities on population health, including both acute exposures, such as deadly infections and injuries, and an accumulation of adverse exposures over the life course, such as nutrition, environmental contaminants, morbidities, and health behaviors.^12^ Results should be examined together with current information on risk factors not yet reflected in life expectancy.

## Methods

### Data

This study used publicly accessible secondary aggregate data from the WHO and UN. These activities do not meet the regulatory definition of human participant research. As such, institutional review board approval and informed consent were not required in accordance with 45 CFR §46. This study followed the Strengthening the Reporting of Observational Studies in Epidemiology (STROBE) guidelines for reporting cross-sectional studies.

Data on number of deaths by cause and age came from the World Health Organization’s (WHO) Global Health Estimates. To obtain cause and age-specific mortality rates, fractions of deaths from each cause by age were multiplied by the all-cause age-specific mortality rate from the United Nations (UN) World Population Prospects (WPP) 2024 (see eAppendix 1 in Supplement 1).^5,13,14^ The I-8 priority conditions were: (1) neonatal conditions; (2) lower respiratory infections; (3) diarrheal diseases; (4) HIV/AIDS; (5) tuberculosis; (6) malaria (7) childhood-cluster diseases: whooping cough, diphtheria, measles, and tetanus; and (8) maternal conditions.^7^

The NCD-7 were also highlighted as priority conditions (see eTable 1 in Supplement 1 for details): (1) atherosclerotic cardiovascular diseases (CVD), (2) hemorrhagic stroke, (3) NCDs strongly linked to infections, (4) NCDs strongly linked to tobacco use, (5) diabetes, (6) road injury, and (7) suicide. The remaining 18 causes were referred to as other causes.

### Statistical Analysis

We used regional classifications from the third *Lancet* Commission on Investing in Health (CIH), consisting of 7 geographic regions (eTable 2 in Supplement 1) and the 3 most populous countries in 2019: China, India, and the US. We focus on 2019 to avoid distortions due to COVID-19. We decomposed the difference in life expectancy at birth between the North Atlantic and other regions and countries into components (in terms of years) attributable to the 33 causes of death. The North Atlantic was chosen since it had the greatest life expectancy (82 years^5^) of the regions highlighted here, and has had high living standards and universal health coverage over a long period.

We used the Pollard decomposition method^15,16^ to quantify the contribution of each cause to the total life expectancy gap and provide 95% uncertainty bounds (UB) obtained from 1000 Monte Carlo simulations (eAppendix 2 in Supplement 1). We present detailed results for the 6 other CIH regions and 3 most populous countries as well as a summary for 165 countries with at least 2 years’ lower life expectancy than the North Atlantic. Analyses were done using Stata version 16 (StataCorp).

We also provide estimates for all causes, regions, and countries in the years 2000, 2010, 2019, and 2021 by sex and overall (eTables 3-6 in Supplement 1 and eTable in Supplement 2). The North Atlantic in 2019 was also used as a benchmark when analyzing other years in the target locations to facilitate comparisons across time. We also show results after removing causes that contributed negatively to the life expectancy gap, which occurred when the target location had achieved lower cause-specific mortality rates than the North Atlantic (eTable 7 in Supplement 1).

We performed 3 sensitivity analyses. First, we estimated results using the Arriaga^17^ decomposition method (eAppendix 3 and eTable 8 in Supplement 1). Second, we decomposed the differences in life expectancy between 10 deciles of roughly equal population size (excluding India, China, and the US) according to life expectancy at birth (eTable 9 in Supplement 1). Third, we decomposed the difference in life expectancy between diverse countries for which the WHO assesses it has high or medium quality data^18^ and the North Atlantic (where data quality was generally of high quality: eTable 10 in Supplement 1).

## Results

### The Overall Contributions of the NCD-7 and I-8 to Life Expectancy Gaps

**Figure 1.**
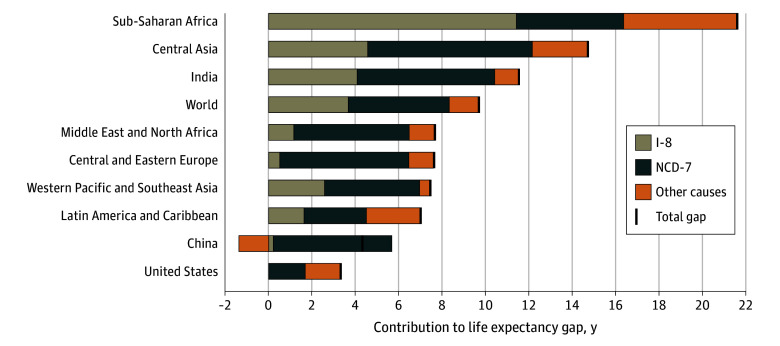
Contributions of Priority Conditions to Life Expectancy Gaps Relative to the North Atlantic, 2019 Comparisons were made with the 2019 North Atlantic (which had a life expectancy of 82 years). The I-8 are neonatal conditions, lower respiratory infections, diarrheal diseases, HIV/AIDS, tuberculosis, malaria, childhood-cluster diseases, and maternal conditions. The NCD-7 are atherosclerotic cardiovascular diseases, hemorrhagic stroke, NCDs strongly linked to infections, NCDs strongly linked to tobacco use, diabetes, road injury, and suicide. Negative contribution suggests that the gap in life expectancy would have been greater had it not been for lower mortality than in the North Atlantic. Therefore, the contribution of some causes or groups of causes can be greater than the total life expectancy gap. I-8 indicates 8 priority infectious and maternal and child health conditions; NCD-7, 7 priority noncommunicable diseases and injuries.

In 2019, sub-Saharan Africa had a 21.61-year lower life expectancy than the North Atlantic benchmark, of which 11.40 (95% UB, 10.92-11.76) years were attributable to the I-8 and 4.96 (95% UB, 4.75-5.02) to the NCD-7 (Figure 1, Table for tabulated estimates). Central Asia had a life expectancy gap of 14.73 years, with 4.53 (95% UB, 4.17-4.97) years due to higher mortality from I-8 and 7.63 (95% UB, 7.11-8.05) years due to the NCD-7. The life expectancy gap in India was 11.54 years, of which 4.05 (95% UB, 3.75-4.49) years were attributable to the I-8 and 6.35 (95% UB, 5.94-6.75) to the NCD-7.

**Table.** Contributions of Causes of Death to Life Expectancy Gaps Relative to the North Atlantic, 2019^a^

Causes of death	Years, No. (% of total gap) [95% UB]								
	China	India	United States	Central Asia	Central and Eastern Europe	Latin America and Caribbean	Middle East and North Africa	Sub-Saharan Africa	Western Pacific and Southeast Asia
Total life expectancy gap	4.32	11.54	3.31	14.73	7.61	7.01	7.67	21.61	7.45
Total contribution of NCD-7	5.48 (127) [4.95 to 6.03]	6.35 (55) [5.94 to 6.75]	1.62 (49) [1.29 to 1.91]	7.63 (52) [7.11 to 8.05]	5.91 (78) [5.76 to 6.05]	2.85 (41) [2.75 to 2.94]	5.30 (69) [5.04 to 5.49]	4.96 (23) [4.75 to 5.02]	4.39 (59) [4.19 to 4.56]
Atherosclerotic CVDs	1.88 (43) [1.60 to 2.17]	2.47 (21) [2.27 to 2.65]	0.72 (22) [0.50 to 0.92]	4.29 (29) [3.95 to 4.55]	4.59 (60) [4.45 to 4.69]	1.20 (17) [1.14 to 1.26]	3.58 (47) [3.38 to 3.71]	1.49 (7) [1.39 to 1.53]	1.41 (19) [1.30 to 1.51]
Diabetes	0.13 (3) [0.11 to 0.16]	0.59 (5) [0.54 to 0.63]	0.19 (6) [0.17 to 0.22]	0.70 (5) [0.64 to 0.77]	0.11 (2) [0.11 to 0.12]	0.84 (12) [0.82 to 0.86]	0.55 (7) [0.52 to 0.59]	0.80 (4) [0.77 to 0.82]	0.48 (6) [0.45 to 0.50]
Infection-related NCDs	0.67 (15) [0.60 to 0.73]	0.55 (5) [0.51 to 0.62]	−0.01 (<.01) [−0.02 to 0]	0.88 (6) [0.82 to 0.96]	0.32 (4) [0.31 to 0.33]	0.25 (4) [0.24 to 0.26]	0.59 (8) [0.56 to 0.63]	0.81 (4) [0.79 to 0.83]	0.65 (9) [0.63 to 0.68]
Tobacco-related NCDs	1.20 (28) [1.01 to 1.40]	1.56 (14) [1.39 to 1.75]	0.27 (8) [0.19 to 0.35]	0.53 (4) [0.41 to 0.68]	0.08 (1) [0.06 to 0.11]	−0.07 (−1) [−0.09 to −0.05]	0.05 (1) [0.01 to 0.10]	−0.08 (<.01) [−0.10 to −0.05]	0.29 (4) [0.24 to 0.33]
Hemorrhagic stroke	1.31 (30) [1.17 to 1.46]	0.69 (6) [0.62 to 0.76]	0.05 (2) [0.04 to 0.07]	0.93 (6) [0.84 to 1.03]	0.39 (5) [0.38 to 0.41]	0.32 (5) [0.31 to 0.34]	0.31 (4) [0.28 to 0.34]	1.06 (5) [1.01 to 1.09]	1.19 (16) [1.12 to 1.25]
Road injury	0.35 (8) [0.33 to 0.36]	0.33 (3) [0.31 to 0.34]	0.24 (7) [0.23 to 0.24]	0.31 (2) [0.28 to 0.32]	0.20 (3) [0.19 to 0.20]	0.35 (5) [0.34 to 0.35]	0.37 (5) [0.35 to 0.37]	0.76 (4) [0.71 to 0.75]	0.39 (5) [0.37 to 0.39]
Suicide	−0.06 (−1) [−0.07 to −0.04]	0.17 (1) [0.15 to 0.19]	0.16 (5) [0.16 to 0.17]	−0.01 (<.01) [−0.03 to 0.01]	0.21 (3) [0.21 to 0.22]	−0.04 (−1) [−0.04 to −0.03]	−0.16 (−2) [−0.16 to −0.15]	0.12 (1) [0.11 to 0.13]	−0.01 (<.01) [−0.02 to 0]
Total contribution of I-8	0.22 (5) [0.16 to 0.28]	4.05 (35) [3.75 to 4.49]	0.05 (1) [0.02 to 0.08]	4.53 (31) [4.17 to 4.97]	0.52 (7) [0.51 to 0.55]	1.65 (24) [1.60 to 1.69]	1.16 (15) [1.08 to 1.27]	11.40 (53) [10.92 to 11.76]	2.54 (34) [2.44 to 2.65]
Childhood-cluster diseases	0.01 (<.01) [0.01 to 0.02]	0.13 (1) [0.08 to 0.26]	0 [0 to 0]	0.28 (2) [0.20 to 0.49]	0 [0 to 0]	0.01 (<.01) [0.01 to 0.02]	0.05 (1) [0.03 to 0.09]	0.45 (2) [0.39 to 0.60]	0.07 (1) [0.06 to 0.10]
Diarrheal diseases	0 [0 to 0.01]	1.17 (10) [0.98 to 1.48]	0.01 (<.01) [0.01 to 0.01]	0.58 (4 ) [0.49 to 0.73]	−0.01 (<.01) [−0.01 to −0.01]	0.09 (1) [0.09 to 0.10]	0.05 (1) [0.04 to 0.09]	1.47 (7) [1.39 to 1.60]	0.31 (4) [0.29 to 0.36]
HIV/AIDS	0.05 (1) [0.05 to 0.06]	0.08 (1) [0.07 to 0.10]	0.03 (1) [0.03 to 0.03]	0.12 (1) [0.10 to 0.22]	0.27 (4) [0.26 to 0.27]	0.17 (2) [0.17 to 0.18]	0.02 (<.01) [0.02 to 0.02]	1.67 (8) [1.60 to 1.71]	0.17 (2) [0.16 to 0.18]
Lower respiratory infections	0.03 (1) [−0.01 to 0.07]	0.78 (7) [0.68 to 0.89]	−0.07 (−2) [−0.09 to −0.05]	0.69 (5) [0.57 to 0.81]	0.14 (2) [0.13 to 0.16]	0.81 (12) [0.77 to 0.84]	0.37 (5) [0.33 to 0.44]	2.18 (10) [2.04 to 2.30]	0.69 (9) [0.64 to 0.74]
Malaria	0 [NA]	0.02 (<.01) [0.02 to 0.03]	0 [NA]	0.01 (<.01) [0 to 0.01]	0 [NA]	0 [0 to 0]	0.01 (<.01) [0.01 to 0.02]	1.32 (6) [1.16 to 1.49]	0.01 (<.01) [0.01 to 0.02]
Maternal conditions	0 [0 to 0]	0.05 (<.01) [0.05 to 0.05]	0.01 (<.01) [0 to 0.01]	0.21 (1) [0.18 to 0.25]	0 [0 to 0]	0.04 (1) [0.04 to 0.04]	0.02 (<.01) [0.02 to 0.03]	0.61 (3) [0.57 to 0.65]	0.06 (1) [0.05 to 0.07]
Neonatal conditions	0.08 (2) [0.04 to 0.12]	1.16 (10) [0.93 to 1.33]	0.08 (2) [0.06 to 0.09]	2.09 (14) [1.73 to 2.38]	0.02 (<.01) [0 to 0.03]	0.43 (6) [0.39 to 0.46]	0.56 (7) [0.49 to 0.62]	1.60 (7) [1.41 to 1.74]	0.65 (9) [0.56 to 0.72]
Tuberculosis	0.04 (1) [0.04 to 0.05]	0.66 (6) [0.61 to 0.70]	0 [0 to 0]	0.55 (4) [0.51 to 0.57]	0.11 (1) [0.10 to 0.11]	0.09 (1) [0.09 to 0.09]	0.07 (1) [0.07 to 0.07]	2.11 (10) [2.00 to 2.15]	0.58 (8) [0.55 to 0.59]
Total contribution of other causes	−1.38 (−32) [−1.94 to −0.84]	1.14 (10) [0.50 to 1.69]	1.64 (50) [1.33 to 1.99]	2.57 (17) [1.94 to 3.20]	1.18 (15) [1.02 to 1.34]	2.51 (36) [2.40 to 2.64]	1.21 (16) [0.98 to 1.49]	5.25 (24) [4.94 to 5.84]	0.53 (7) [0.29 to 0.77]

Abbreviations: CVD, cardiovascular diseases; I-8, 8 priority infectious and maternal and child health conditions; NCD-7, 7 priority noncommunicable diseases and injuries.

^a^ Comparisons were made to the 2019 North Atlantic (which had a life expectancy of 82 years). Negative contribution suggests that the gap in life expectancy would have been greater had it not been for lower mortality from that cause than in the North Atlantic. Therefore, the contribution of some causes or groups of causes can be over 100% of the total life expectancy gap.

In 2019, the life expectancy gap was 7.45 years in Western Pacific and Southeast Asia, with 2.54 (95% UB, 2.44-2.65) years explained by the I-8 and 4.39 (95% UB, 4.19-4.56) by the NCD-7. Latin America and the Caribbean had a 7.01-year gap, with 1.65 (95% UB, 1.60-1.69) years due to the I-8 and 2.85 (95% UB, 2.75-2.94) due to the NCD-7. The gap in the Middle East and North Africa was 7.67 years, of which 1.16 (95% UB, 1.08-1.27) years were attributable to the I-8 and 5.30 (95% UB, 5.04-5.49) to the NCD-7.

In Central and Eastern Europe, the gap was 7.61 years, of which 0.52 (95% UB, 0.51 to 0.55) years were accounted for by the I-8 and 5.91 (95% UB, 5.76 to 6.05) years by the NCD-7. The life expectancy gap in China was 4.32 years, with 0.22 (95% UB, 0.16 to 0.28) years attributable to the I-8 and 5.48 (95% UB, 4.95 to 6.03) years to the NCD-7. Mortality from causes not included in the priority conditions (other causes) was lower in China than the North Atlantic, leading the life expectancy gap to be 1.38 (95% UB, −1.94 to −0.84) years lower than it would have otherwise been. Therefore, the contribution of the NCD-7 was greater than the total life expectancy gap. In the US, the life expectancy gap was 3.31 years, with 0.05 (95% UB, 0.02 to 0.08) years due to the I-8 and 1.62 (95% UB, 1.29 to 1.91) years due to the NCD-7.

### Contribution of Specific Priority Conditions to Gaps in Life Expectancy

**Figure 2.**
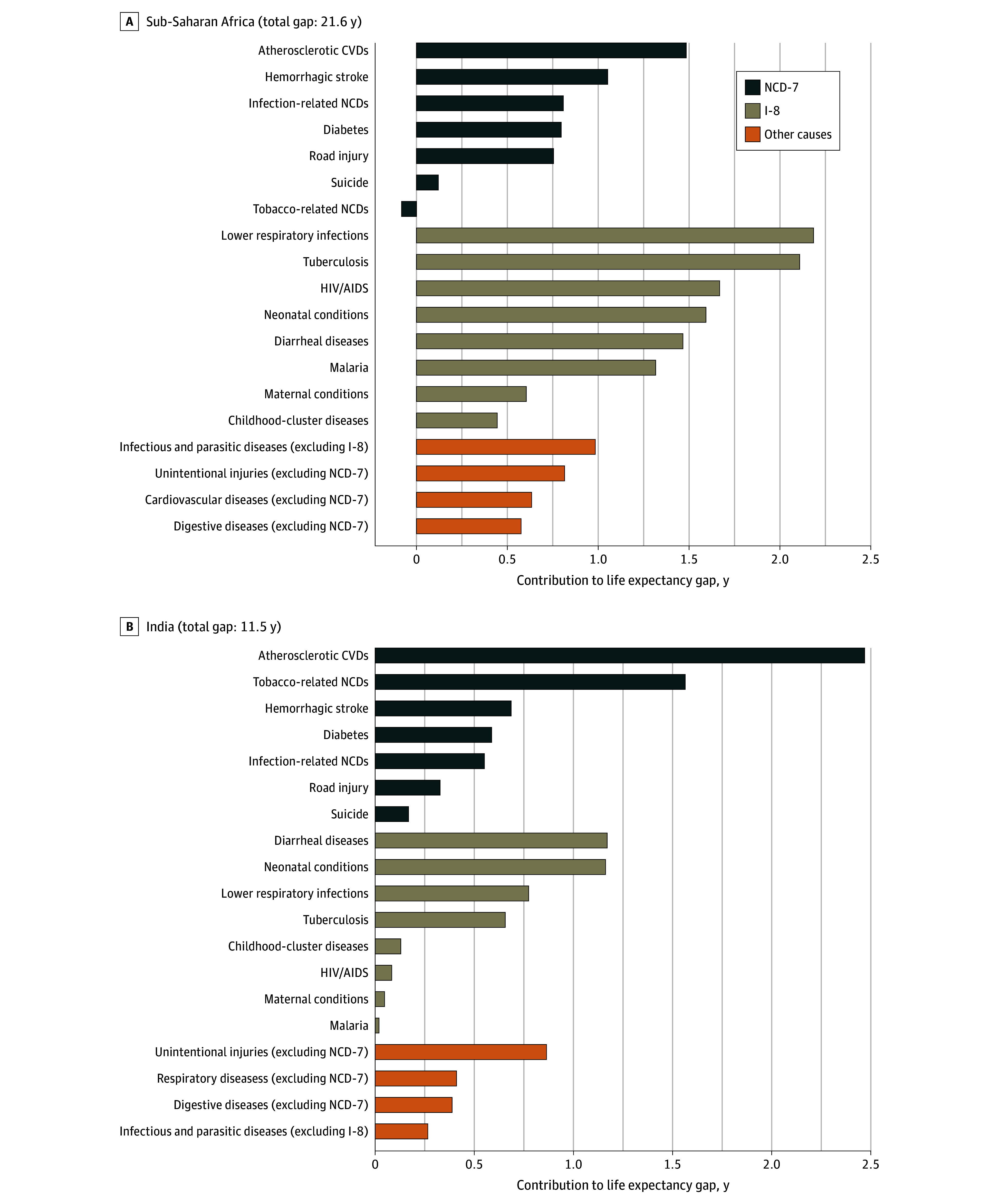
Contributions of Causes of Death to Life Expectancy Gaps Relative to the North Atlantic, 2019 Top 4 other causes are shown (positive contributions only). Comparisons were made with the North Atlantic (which had a life expectancy of 82 years). Negative contribution suggests that the gap in life expectancy would have been greater had it not been for lower mortality than in the 2019 North Atlantic. Therefore, the contribution of some causes or groups of causes can be greater than the total life expectancy gap. CVD indicates cardiovascular disease; I-8, 8 priority infectious and maternal and child health conditions; NCD-7, 7 priority noncommunicable diseases and injuries.

In sub-Saharan Africa, in 2019, lower respiratory infections accounted for the largest share by individual cause, 2.18 (95% UB, 2.04-2.30) years, or 10% of the total gap (Figure 2, Table for tabulated estimates). Tuberculosis accounted for 2.11 (95% UB, 2.00-2.15) years (10% of the total gap), HIV/AIDS for 1.67 (95% UB, 1.60-1.71) years (8% of the total gap), neonatal conditions for 1.60 (95% UB, 1.41-1.74) years (7% of the total gap), atherosclerotic CVDs for 1.49 (95% UB, 1.39-1.53) years (7% of the total gap), and diarrheal diseases for 1.47 (95% UB, 1.39-1.60) years (7% of the total gap).

**Figure 3.**
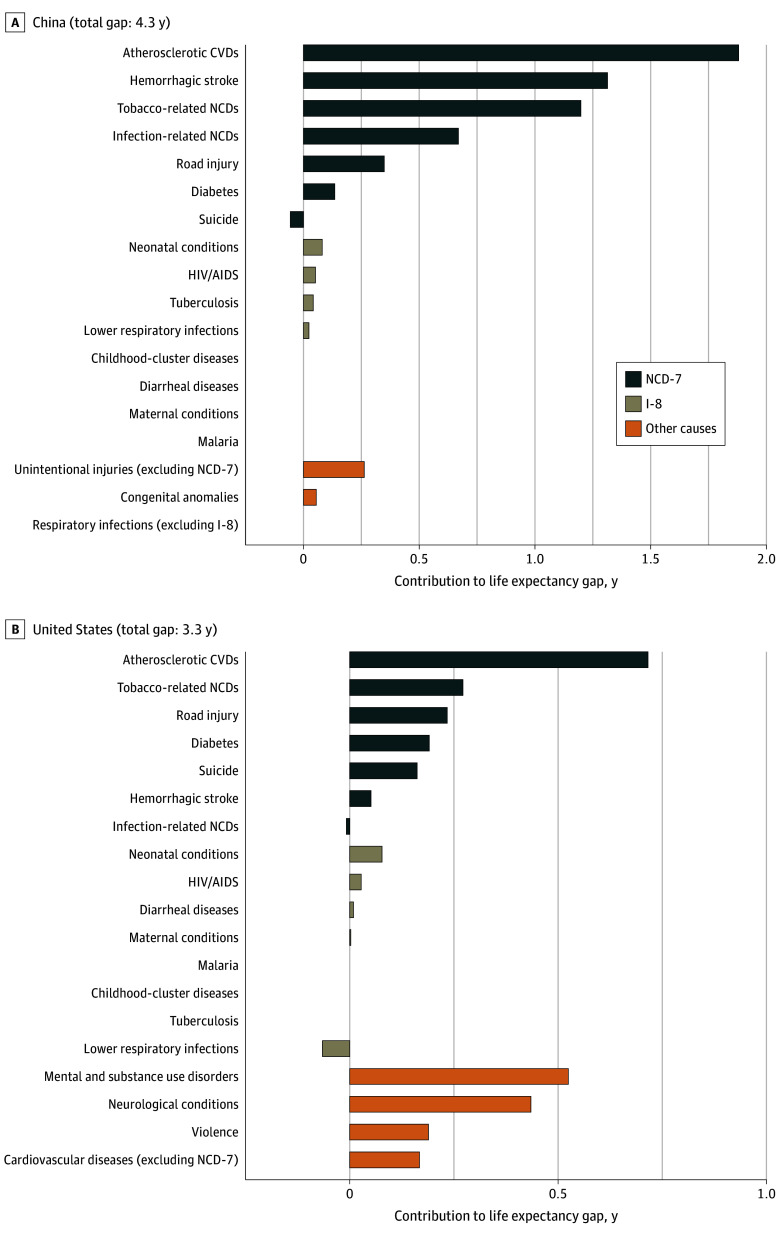
Contributions of Causes of Death to Life Expectancy Gaps Relative to the North Atlantic, 2019 Top 4 other causes are shown (positive contributions only). Only 3 other causes had a positive contribution in China. Comparisons were made with the North Atlantic (which had a life expectancy of 82 years). Negative contribution suggests that the gap in life expectancy would have been greater had it not been for lower mortality than in the 2019 North Atlantic. Therefore, the contribution of some causes or groups of causes can be greater than the total life expectancy gap. CVD indicates cardiovascular disease; I-8, 8 priority infectious and maternal and child health conditions; NCD-7, 7 priority noncommunicable diseases and injuries.

In India, diarrheal diseases accounted for 1.17 (95% UB, 0.98-1.48) years (10% of the total gap) and neonatal conditions for 1.16 (95% UB, 0.93-1.33) years of the 11.54-year life expectancy gap (10% of the total gap). Tuberculosis and lower respiratory infections accounted for around 6% to 7% each, contributing 0.66 (95% UB, 0.61-0.70) and 0.78 (95% UB, 0.68-0.89) years, respectively. However, in India, atherosclerotic CVDs accounted for the largest share of the life expectancy gap, 2.47 (95% UB, 2.27-2.65) years (21% of the total gap), followed by tobacco-related NCDs, 1.56 (95% UB, 1.39-1.75) years (14%). Hemorrhagic stroke, diabetes, and infection-related NCDs each contributed approximately 5% to 6% each to the total gap in India. In China, atherosclerotic CVDs explained 1.88 (95% UB, 1.60-2.17) years, or 43% of the life expectancy gap (Figure 3); hemorrhagic stroke accounted for 1.31 (95% UB, 1.17-1.46) years (30% of the total gap), tobacco-related NCDs for 1.20 (95% UB, 1.01-1.40) years (28% of the total gap), and infection-related NCDs for 0.67 (95% UB, 0.60-0.73) years (15% of the total gap). Atherosclerotic CVDs accounted for the largest share of the life expectancy gap in all regions except sub-Saharan Africa (eFigures 1-3 in Supplement 1).

### Most Important Causes Not Included in the Priority Conditions

Infectious and parasitic diseases other than those included in the I-8 contributed considerably to the life expectancy gap for sub-Saharan Africa (Figure 2). In the US, mental and substance use disorders and neurological conditions (eg, dementias) contributed substantially to the life expectancy gap in relative terms (Figure 3). Unintentional injuries other than road injuries contributed at least somewhat to the life expectancy gap in most regions. Violence contributed considerably to the life expectancy deficit for Latin America and the Caribbean (eFigure 2 in Supplement 1).

### Percentage Gap Accounted for by Priority Conditions Across Countries

**Figure 4.**
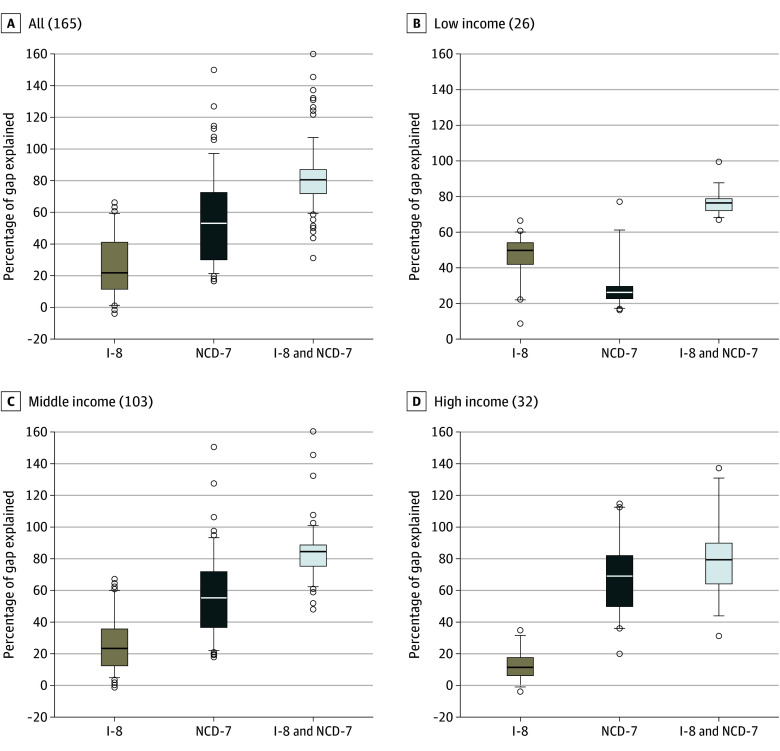
Percentage of Life Expectancy Gap Compared With the North Atlantic Attributable to Priority Conditions: Distribution Across Countries, 2019 Number of countries is shown in parentheses. Only countries with at least 2 years’ lower life expectancy than the North Atlantic (which had a life expectancy of 82 years) were included. Results are shown overall and by 2019 World Bank Income groups (4 countries were not classified). Percentiles 5 and 95 (line) and 25, 50, and 75 (box) are shown. Dots indicate country-estimates below percentile 5 and above percentile 95. Countries were equally weighted for descriptive statistics. Negative contribution and contribution over 100% suggest that the gap in life expectancy would have been greater had it not been for lower mortality for some causes than in the 2019 North Atlantic. See eTable 11 in Supplement 1 for tabulated estimates. The I-8 are neonatal conditions, lower respiratory infections, diarrheal diseases, HIV/AIDS, tuberculosis, malaria, childhood-cluster diseases, and maternal conditions. The NCD-7 are atherosclerotic cardiovascular diseases, hemorrhagic stroke, NCDs strongly linked to infections, NCDs strongly linked to tobacco use, diabetes, road injury, and suicide. I-8 indicates 8 priority infectious and maternal and child health conditions; NCD-7, 7 priority noncommunicable diseases and injuries.

The percentage share accounted for by both the I-8 and NCD-7 combined ranged from 31% to 160% across the 165 countries that had at least 2 years’ lower life expectancy than the North Atlantic, and was 80% (IQR, 71-88) in the median country (Figure 4; eTable 11 in Supplement 1 for tabulated estimates). Only in low-income countries was the median percentage share accounted for by the I-8 greater than the NCD-7. The cross-country correlation of the total gap in life expectancy with the share accounted for by the I-8 was large and positive; large and negative for the NCD-7; and low for the I-8 and NCD-7 combined (eFigure 4 in Supplement 1).

### Changes Over Time

Mortality from the I-8 accounted for 21.42 (95% UB, 20.57-22.19) years of a 31.14-year life expectancy gap between sub-Saharan Africa in the year 2000 and the 2019 North Atlantic benchmark. The life expectancy gap between the (2019) North Atlantic and sub-Saharan Africa declined by almost 10 years between 2000 and 2019, entirely due to a decline in the estimated impact of the I-8 on life expectancy; particularly HIV/AIDS, diarrheal diseases, malaria, tuberculosis, and childhood cluster diseases (eTable 3 in Supplement 1; Table; eTable 4 in Supplement 1 for UBs). Meanwhile, the estimated impact of the NCD-7 on life expectancy, especially atherosclerotic CVDs and to a lesser extent diabetes, increased somewhat in sub-Saharan Africa.

India transitioned from having the majority of the life expectancy gap accounted for by the I-8 in 2000 (11.87; 95% UB, 10.98-12.98 years of a 19.60-year life expectancy gap) to having most of it accounted for by the NCD-7 in 2019; diarrheal diseases, tuberculosis, neonatal conditions, and lower respiratory infections had the most significant decline in estimated impact on life expectancy in India, while the estimated impact of atherosclerotic CVDs and diabetes on life expectancy rose somewhat. Conversely, there were considerable declines in the estimated impact of atherosclerotic CVDs on life expectancy in the US, Central and Eastern Europe, Middle East and North Africa, and Latin America and the Caribbean. China had a substantial decline in the estimated impact of tobacco-related NCDs, hemorrhagic stroke, and infection-related NCDs on life expectancy, while the estimated impact of atherosclerotic CVDs on life expectancy increased somewhat. The contribution of causes not considered priority conditions to the life expectancy gap relative to the 2019 North Atlantic rose in the US, mostly due to a rise in the contribution of mental and substance use disorders. In China, the estimated impact of these other causes on life expectancy declined considerably, such that in 2019 they suppressed the life expectancy gap (ie, the gap in life expectancy for China would have been greater had it not been for lower mortality from these conditions than in the North Atlantic).

The North Atlantic had a 0.38-year decline in life expectancy between 2019 and 2021, entirely accounted for by COVID-19 (eTable 5 in Supplement 1). Life expectancy in all regions except China was impacted by COVID-19 to a greater extent than in the North Atlantic, increasing the life expectancy gaps.

### Differences by Sex

The I-8 contributed more to the life expectancy gap for female individuals than male individuals in India, primarily due to a greater contribution of diarrheal diseases and to a lesser extent due to lower respiratory infections and neonatal conditions (eTable 6 in Supplement 1). Maternal conditions also contributed to the life expectancy gap for female individuals in some regions, such as sub-Saharan Africa and Central Asia.

Overall, the life expectancy gap was much greater for male than female individuals in Central and Eastern Europe when compared with male and female individuals in the North Atlantic, respectively. Tobacco-related NCDs contributed somewhat more to the life expectancy gap for male individuals in Central Asia. Violence had some impact on life expectancy for male individuals in the US and a large impact in Latin America and the Caribbean.

### Results From Sensitivity Analyses

Results look similar when using the Arriaga decomposition method instead of Pollard (eTable 8 in Supplement 1). Decomposing the difference in life expectancy between the top decile in terms of life expectancy and other deciles also suggests that the 15 priority conditions account for the majority of the gap, usually 70% to 80% (eTable 9 in Supplement 1). Decomposing the life expectancy gap between the North Atlantic and 9 countries with high or medium quality data suggest the priority conditions account for close to or above 80% of the difference (eTable 10 in Supplement 1).

## Discussion

In the 6 regions and the 3 most populous countries highlighted in this cross-sectional study, mortality from the 15 priority conditions, the I-8 and NCD-7, together accounted for over 80% of the life expectancy disparity relative to the North Atlantic in most cases. The NCD-7 commonly accounted for over half the total gap in 2019. We observed an impressive decline in the estimated impact of the I-8 on life expectancy in sub-Saharan Africa from 2000 to 2019, which drove a large overall decline in the life expectancy disparity. Meanwhile, the estimated impact of the NCD-7 on life expectancy rose. Furthermore, India transitioned from having most of the disparity attributable to deaths from the I-8 in 2000 to having a larger share explained by the NCD-7 in 2019.

Among the 18 causes not among the I-8 and NCD-7, few contributed substantially to the life expectancy gap—despite being broadly defined—which underscores the importance of relatively few priority conditions. However, violence contributed to the life expectancy gap for male individuals in Latin America and the Caribbean compared with male individuals in the North Atlantic, and drug use disorder contributed to the life expectancy gap for the US. However, by 2021, the COVID-19 pandemic had put most regions further behind the North Atlantic.

Our results highlight the importance of ensuring coverage of the most essential health interventions aimed at the 15 priority conditions. The WHO suggested that in 2021, 4.5 billion people were not covered by essential health services^19,20^ and that current health expenditure is far below what is needed to provide these.^6,21,22^ The World Bank estimated that even with favorable policies and economic growth, the financing gap in low and middle-income countries would only be reduced by about a third by 2030.^6^ In settings with severely constrained finances, covering a limited number of critical interventions addressing the most urgent health problems might be more financially and administratively feasible without sacrificing population health gains.^7^

The priority conditions are prominent or rising health concerns with known determinants and cost-effective solutions.^7,23^ The third edition of the Disease Control Priorities suggested 21 packages of essential interventions grouped according to relevance to a particular professional community.^23^ The relative contribution of each cause to life expectancy disparities can help guide policy emphasis, planning, and the allocation of additional health spending across these packages. Of the causes highlighted in this study, atherosclerotic CVDs accounted for the largest share of the life expectancy gap in all regions except sub-Saharan Africa (where it also accounts for a substantial and increasing share). The importance of atherosclerotic CVDs highlights the need for improving diagnosis of hypertension^24^ and medical interventions (eg, statins) and behavioral interventions (eg, reduced smoking, improved diet, and increased physical activity) to delay mortality from these causes.^25^ Preventive interventions for diabetes, such as taxation of sugary drinks^26^ and improved diagnosis and treatment,^27^ can be implemented to combat the considerable and rising relative estimated impact of diabetes on life expectancy in some regions.

The significant contribution of tobacco-related NCDs to the life expectancy gaps for China and India relative to the North Atlantic suggests that gains can be made through tobacco control, for instance by restricting smoking^28^ and taxation.^9^ However, in both China and India, smoking is much more prevalent among male individuals (while both prevalence and sex differences are smaller in the North Atlantic). Still, the contribution of tobacco-related NCDs to the life expectancy gap was similar for male and female individuals in China and greater for female individuals in India, which may suggest that other factors, such as outdoor^29^ and indoor^30^ air pollution, may play a role.

An expansion of maternal and child health interventions (especially prevention of diarrhea, neonatal conditions, and lower respiratory infections) and tuberculosis interventions remains essential in sub-Saharan Africa, India, Central Asia, and Western Pacific and Southeast Asia. Since childhood deaths often result from repeated adverse exposures, an intervention aimed at reducing deaths from one condition can also indirectly reduce mortality from other causes.^31^ Poor health in childhood can also have compounding effects on human development in terms of health,^32,33^ physiological^34^ and cognitive development,^35^ and schooling and income,^36^ which in turn are linked to life expectancy.^37^

Some of the causes highlighted here contributed little to the life expectancy disparities. The relatively minor contribution of childhood-cluster diseases highlights the success of the widespread (although incomplete) distribution of vaccines.^38,39^ Furthermore, suicide contributed little (or, often, nothing) to life expectancy disparities since suicides are not particularly rare in the North Atlantic.^40^ Suicide is also only the most extreme consequence of poor mental health, which is a rising health concern, and is associated with heightened mortality from a range of conditions, likely stemming from factors such as health behaviors, access to care, and socioeconomic factors.^41,42^

Previous studies have used the measures of amenable mortality—or deaths that could have been avoided with timely and effective care—to measure shortcomings in health.^43,44^ Amenable mortality evidences a similar geographic distribution as the life expectancy disparities.^43^ Our study takes a more general approach using a more straightforward metric and benchmarks shortcomings to an outcome that has been achieved. However, studies using amenable mortality remind us that further improvements can be achieved even in our benchmark—the North Atlantic—by reducing avoidable deaths.

### Limitations

Some limitations and caveats should be kept in mind when interpreting these results. First, there are caveats related to using life expectancy to identify current shortcomings in health. Mortality often results from repeated or extended periods of adverse exposures (eg, smoking, diet, and early life infections and undernutrition) involving many interlinked factors. Since (period) life expectancy gives a snapshot of current age-specific mortality, the decomposition will not capture recent changes in underlying risk factors that may impact mortality rates with delays. For example, recent dramatic declines in tobacco use in India are likely to reduce the impact of tobacco-related NCDs in the future.^45^ Therefore, the results from this study should be viewed together with information on recent changes in underlying risk factors.

Second, there are limitations related to data quality (see further discussion in eAppendix 1 in Supplement 1). Data in countries without well-functioning vital registration systems relied on various methods and sources—such as verbal autopsies and modeled estimates from Global Burden of Disease 2021.^14,46^ For example, death registration data were completely unavailable or unusable for 74 countries, including large countries such as India and China and most of sub-Saharan Africa.^18^ Cause of death coding practices may also vary across countries and over time.^47,48,49^ However, data quality issues were unlikely to be an underlying factor behind our conclusions since the importance of the priority conditions was also apparent when only including countries deemed to have high or medium quality data.

Third, our grouping of causes of deaths according to important risk factors needs to consider local contexts. For example, in many countries, air pollution may contribute substantially to the groups of causes that are associated with smoking. Furthermore, categorization according to risk factors such as smoking and infections only included the major causes while other relevant causes of deaths were excluded: for example, smoking also increases the risk of esophageal, colon, and bladder cancers^50,51^ and chronic salmonella also increases the risk of gall bladder cancer.^52^

Additionally, there are important considerations to keep in mind when combining mortality data from the UN WPP and the WHO. The total number of deaths by age from the 2 sources tend to be similar: the WHO tends to estimate fewer deaths than the UN for ages 1 to 50 years and more deaths at age 85 years and older. However, there are country specific differences that are worth noting, particularly concerning changes over time: countries in West Africa, particularly the Central African Republic, had large increases in the number of deaths in 2019 according to the UN WPP data while no such increase was observed in the WHO data. Therefore, the estimated impact of most causes on life expectancy will increase in absolute terms in 2019 when compared with earlier years in these countries, since we rely on fraction of deaths by cause from the WHO and all-cause mortality rates from the UN WPP. However, our main interest was comparing global regions and countries at a single point in time: We also provide estimates for several years, which all show the overwhelming importance of the priority conditions.

## Conclusions

This cross-sectional study underscores the significant contribution of 15 priority conditions to life expectancy disparities, with notable regional variations and transitions over time. The decline in death rate from the I-8, particularly in sub-Saharan Africa, contrasts with the rising importance of the NCD-7, highlighting the evolving nature of global health challenges. These findings emphasize the critical importance of focusing limited resources on the key conditions that contribute the most to poor health and health disparities.

## Notes

Supplement 1. **eAppendix 1.** More Information on Data **eTable 1.** Causes of Death According to the World Health Organization’s Global Health Estimates (GHE) Included in the I-8 and NCD-7 Conditions **eTable 2.** Regions **eAppendix 2. **Pollard Decomposition Method **eTable 3. **Contributions of Causes of Death to Life Expectancy Gaps Relative to the 2019 North Atlantic: All Causes in 2000 and 2019 **eTable 4. **Contributions of Causes of Death to Life Expectancy Gaps Relative to the 2019 North Atlantic: Results for the Year 2000 **eTable 5.** Contributions of Causes of Death to Life Expectancy Gaps Relative to the 2019 North Atlantic: Results for the Year 2021 **eTable 6.** Contributions of Causes of Death to Life Expectancy Gaps Relative to the North Atlantic, 2019: All Causes for Males (M) and Females (F) **eTable 7.** Contributions of Causes of Death to Life Expectancy Gaps Relative to the North Atlantic, 2019: Removing Negative Contributions **eAppendix 3.** Arriaga's Decomposition Method **eTable 8.** Contributions of Causes of Death to Life Expectancy Gaps Relative to the North Atlantic, 2019: Arriaga's Method **eTable 9. **Contributions of Causes of Death to Life Expectancy Gaps Relative to the Top Life Expectancy Decile, 2019 **eTable 10.** Contributions of Causes of Death to Life Expectancy Gaps Relative to the North Atlantic, 2019: Selected Countries with High or Medium Quality Data **eFigure 1.** Contributions of Causes of Death to Life Expectancy Gaps Relative to the North Atlantic: Central Asia and Western Pacific and Southeast Asia, 2019 **eFigure 2.** Contributions of Causes of Death to Life Expectancy Gaps Relative to the North Atlantic: Central and Eastern Europe and Latin America and the Caribbean, 2019 **eFigure 3.** Contributions of Causes of Death to Life Expectancy Gaps Relative to the North Atlantic: The Middle East and North Africa and the World, 2019 **eTable 11.** Tabulated Estimates from Figure 4: Percentage of Life Expectancy Gap Compared to the North Atlantic Attributable to Priority Conditions: Distribution Across Countries, 2019 **eFigure 4.** Country-Level Correlation Between the Percentage of Life Expectancy Gap Compared to the 2019 North Atlantic Attributable to the 15 Priority Conditions and the Total Life Expectancy Gap
eReferences.

Supplement 2. **eTable.** Contribution of All Causes to Life Expactancy Gaps Relative to the 2019 North Atlantic: 2000, 2010, 2019, and 2021, for All Countries and Regions, by Sex and Overall

Supplement 3.
Data Sharing Statement
