# Supplementary material for: Priority Health Conditions and Global Life Expectancy Disparities
Source: JAMA Netw Open. 2025 May 23;8(5):e2512198. doi: 10.1001/jamanetworkopen.2025.12198 (PMC12102710; doi:10.1001/jamanetworkopen.2025.12198)
Supplement: Supplement 1. — eAppendix 1. More Information on Data eTable 1. Causes of Death According to the World Health Organization’s Global Health Estimates (GHE) Included in the I-8 and NCD-7 Conditions eTable 2. Regions eAppendix 2. Pollard Decomposition Method eTable 3. Contributions of Causes of Death to Life Expectancy Gaps Relative to the 2019 North Atlantic: All Causes in 2000 and 2019 eTable 4. Contributions of Causes of Death to Life Expectancy Gaps Relative to the 2019 North Atlantic: Results for the Year 2000 eTable 5. Contributions of Causes of Death to Life Expectancy Gaps Relative to the 2019 North Atlantic: Results for the Year 2021 eTable 6. Contributions of Causes of Death to Life Expectancy Gaps Relative to the North Atlantic, 2019: All Causes for Males (M) and Females (F) eTable 7. Contributions of Causes of Death to Life Expectancy Gaps Relative to the North Atlantic, 2019: Removing Negative Contributions eAppendix 3. Arriaga's Decomposition Method eTable 8. Contributions of Causes of Death to Life Expectancy Gaps Relative to the North Atlantic, 2019: Arriaga's Method eTable 9. Contributions of Causes of Death to Life Expectancy Gaps Relative to the Top Life Expectancy Decile, 2019 eTable 10. Contributions of Causes of Death to Life Expectancy Gaps Relative to the North Atlantic, 2019: Selected Countries with High or Medium Quality Data eFigure 1. Contributions of Causes of Death to Life Expectancy Gaps Relative to the North Atlantic: Central Asia and Western Pacific and Southeast Asia, 2019 eFigure 2. Contributions of Causes of Death to Life Expectancy Gaps Relative to the North Atlantic: Central and Eastern Europe and Latin America and the Caribbean, 2019 eFigure 3. Contributions of Causes of Death to Life Expectancy Gaps Relative to the North Atlantic: The Middle East and North Africa and the World, 2019 eTable 11. Tabulated Estimates from Figure 4: Percentage of Life Expectancy Gap Compared to the North Atlantic Attributable to Priority Conditions: Distribution Acro [file jamanetwopen-e2512198-s001.pdf]

Supplemental Online Content

Karlsson O, Chang AY, Norheim OF, Mao W, Bolongaita S, Jamison DT. Priority Health Conditions and Global Life Expectancy Disparities. JAMA Netw Open. 2025;8(5): e2512198. doi:10.1001/jamanetworkopen.2025.12198

eAppendix 1. More Information on Data

eTable 1. Causes of Death According to the World Health Organization's Global Health Estimates (GHE) Included in the I-8 and NCD-7 Conditions

eTable 2. Regions

eAppendix 2. Pollard's Decomposition Method

eTable 3. Contributions of Causes of Death to Life Expectancy Gaps Relative to the 2019 North Atlantic: All Causes in 2000 and 2019

eTable 4. Contributions of Causes of Death to Life Expectancy Gaps Relative to the 2019 North Atlantic: Results for the Year 2000

eTable 5. Contributions of Causes of Death to Life Expectancy Gaps Relative to the 2019 North Atlantic: Results for the Year 2021

eTable 6. Contributions of Causes of Death to Life Expectancy Gaps Relative to the North Atlantic, 2019: All Causes for Males (M) and Females (F)

eTable 7. Contributions of Causes of Death to Life Expectancy Gaps Relative to the North Atlantic, 2019: Removing Negative Contributions

eAppendix 3. Arriaga's Decomposition Method

eTable 8. Contributions of Causes of Death to Life Expectancy Gaps Relative to the North Atlantic, 2019: Arriaga's Method

eTable 9. Contributions of Causes of Death to Life Expectancy Gaps Relative to the Top Life Expectancy Decile, 2019

eTable 10. Contributions of Causes of Death to Life Expectancy Gaps Relative to the North Atlantic, 2019: Selected Countries with High or Medium Quality Data

eFigure 1. Contributions of Causes of Death to Life Expectancy Gaps Relative to the North Atlantic: Central Asia and Western Pacific & Southeast Asia, 2019

eFigure 2. Contributions of Causes of Death to Life Expectancy Gaps Relative to the North Atlantic: Central & Eastern Europe and Latin America & the Caribbean, 2019

eFigure 3. Contributions of Causes of Death to Life Expectancy Gaps Relative to the North Atlantic: The Middle East & North Africa and the World, 2019

eTable 11. Tabulated Estimates from Figure 4: Percentage of Life Expectancy Gap Compared to the North Atlantic Attributable to Priority Conditions: Distribution Across Countries, 2019

eFigure 4. Country-Level Correlation Between the Percentage of Life Expectancy Gap Compared to the 2019 North Atlantic Attributable to the 15 Priority Conditions and the Total Life Expectancy Gap

eReferences

This supplemental material has been provided by the authors to give readers additional information about their work.

© 2025 Karlsson O et al. *JAMA Network Open*.

eAppendix 1. More Information on Data

The World Health Organization's (WHO) Global Health Estimates (GHE) contain the number of deaths by cause for each country, as collected by vital registration systems and death registers when available, or otherwise estimated using various data and methods.[1,2] Deaths were reported by age and sex for 204 countries over the period 2000–2021. Five-year age groups were used for ages 5–84, with an open-ended group for 85 and older. Deaths before age five were estimated for two age groups: 0–11 months and 1–4 years.

In the GHE, causes of deaths are divided into four levels (see page 62 in eReference [3]). The level of detail available varies across countries, with many low- and middle-income countries having zero deaths for some causes at levels three and four, which presumably stems from reporting issues in many cases. Collectively, the 15 priority conditions consist of 26 causes of death as defined in the WHO GHE data (see eTable 1). The 26 causes used to construct the priority conditions are causes at levels two, three, or four and were recorded in almost all countries: Notable exceptions were liver cancer secondary to hepatitis C, for which zero deaths were recorded in Sri Lanka, and cirrhosis due to hepatitis C, for which zero deaths were recorded in Sri Lanka, Kenya, and Senegal. Some causes were expected to have zero deaths in many locations, such as from Malaria.

For the 18 "other causes" we used level two causes (subtracting deaths from the priority conditions when relevant) to ensure coverage of all causes in all countries. Additionally, COVID-19 and pandemic related causes were included for 2021. Two level two causes (oral conditions and sense organ diseases) had no recorded deaths in most low- and middle-income countries: These deaths were few and were divided

© 2025 Karlsson O et al. *JAMA Network Open*.

proportionally among the deaths from other causes in countries where these were recorded.

We multiply the proportion of all deaths from each cause within each age interval from the WHO GHE by the corresponding all-cause age-specific mortality rate from the United Nations (UN) World Population Prospects (WPP) 2024:[4] which is more widely used for life expectancy estimation than the WHO data.

© 2025 Karlsson O et al. *JAMA Network Open*.

eTable 1. Causes of Death According to the World Health Organization's Global Health Estimates (GHE) Included in the I-8 and NCD-7 Conditions

	**I-8**	**GHE causes of death**
1	Childhood-cluster diseases: whooping cough, diphtheria, measles, tetanus	Childhood-cluster diseases
2	Diarrheal diseases	Diarrheal diseases
3	HIV/AIDS	HIV/AIDS
4	Lower respiratory infections	Lower respiratory infections
5	Malaria	Malaria
6	Maternal conditions	Maternal conditions
7	Neonatal conditions	Neonatal conditions
8	Tuberculosis	Tuberculosis
	**NCD-7**	**GHE causes of death**
1	Atherosclerotic CVDs	Ischemic heart disease<br>Ischemic stroke
2	Diabetes	Chronic kidney disease due to diabetes<br>Diabetes mellites
3	Haemorrhagic stroke	Haemorrhagic stroke
4	NCDs strongly linked to infections	Cervical cancer<br>Cirrhosis due to hepatitis B<br>Cirrhosis due to hepatitis C<br>Liver cancer secondary to hepatitis B<br>Liver cancer secondary to hepatitis C<br>Rheumatic heart disease<br>Stomach cancer
5	Road injury	Road injury
6	NCDs strongly linked to tobacco use	Chronic obstructive pulmonary disease<br>Larynx cancer<br>Mouth and oropharynx cancer<br>Trachea, bronchus, and lung cancer
7	Suicide	Self-harm

CVD = cardiovascular disease. NCD = Noncommunicable disease.

© 2025 Karlsson O et al. *JAMA Network Open*.

eTable 2. Regions

Central Asia	Central & Eastern Europe	Latin America & the Caribbean	Middle East & North Africa	North Atlantic	Sub-Saharan Africa	Western Pacific & Southeast Asia
Afghanistan	Albania	Antigua & Barbuda	Algeria	Andorra	Angola	American Samoa
Azerbaijan	Armenia	Argentina	Bahrain	Austria	Benin	Australia
Kazakhstan	Belarus	Bahamas	Egypt	Belgium	Botswana	Bangladesh
Kyrgyzstan	Bosnia & Herzegovina	Barbados	Iran	Bermuda	Burkina Faso	Bhutan
Mongolia	Bulgaria	Belize	Iraq	Canada	Burundi	Brunei Darussalam
Pakistan	Croatia	Bolivia	Israel	Cyprus	Cabo Verde	Cambodia
Tajikistan	Czechia	Brazil	Jordan	Denmark	Cameroon	Cook Islands
Turkmenistan	Estonia	Chile	Kuwait	Finland	Central African Republic	Fiji
Uzbekistan	Georgia	Colombia	Lebanon	France	Chad	Guam
	Hungary	Costa Rica	Libya	Germany	Comoros	Indonesia
	Latvia	Cuba	Morocco	Greece	Congo	Japan
	Lithuania	Dominica	Oman	Greenland	Côte d'Ivoire	Kiribati
	Moldova	Dominican Republic	Palestine	Iceland	DR Congo	Lao
	Montenegro	Ecuador	Qatar	Ireland	Djibouti	Malaysia
	North Macedonia	El Salvador	Saudi Arabia	Italy	Equatorial Guinea	Maldives
	Poland	Grenada	Syria	Luxembourg	Eritrea	Marshall Islands
	Romania	Guatemala	Tunisia	Malta	Eswatini	Micronesia
	Russia	Guyana	Türkiye	Monaco	Ethiopia	Myanmar
	Serbia	Haiti	United Arab Emirates	Netherlands	Gabon	Nauru
	Slovakia	Honduras	Yemen	Norway	Gambia	Nepal
	Slovenia	Jamaica		Portugal	Ghana	New Zealand
	Ukraine	Mexico		San Marino	Guinea	Niue
		Nicaragua		Spain	Guinea-Bissau	North Korea
		Panama		Sweden	Kenya	Northern Mariana Islands
		Paraguay		Switzerland	Lesotho	Palau
		Peru		United Kingdom	Liberia	Papua New Guinea
		Puerto Rico			Madagascar	Philippines
		Saint Kitts & Nevis			Malawi	Samoa
		Saint Lucia			Mali	Singapore
		Saint Vincent & the Grenadines			Mauritania	Solomon Islands
		Suriname			Mauritius	South Korea
		Trinidad & Tobago			Mozambique	Sri Lanka
		US Virgin Islands			Namibia	Taiwan
		Uruguay			Niger	Thailand
		Venezuela			Nigeria	Timor-Leste
					Rwanda	Tokelau

© 2025 Karlsson O et al. *JAMA Network Open*.

Central Asia	Central & Eastern Europe	Latin America & the Caribbean	Middle East & North Africa	North Atlantic	Sub-Saharan Africa	Western Pacific & Southeast Asia
					Sao Tome & Principe	Tonga
					Senegal	Tuvalu
					Seychelles	Vanuatu
					Sierra Leone	Viet Nam
					Somalia	
					South Africa	
					South Sudan	
					Sudan	
					Tanzania	
					Togo	
					Uganda	
					Zambia	
					Zimbabwe	

© 2025 Karlsson O et al. *JAMA Network Open*.

eAppendix 2. Pollard's Decomposition Method

Pollard's decomposition method was used to quantify the contribution of each cause to the total life expectancy gap between the North Atlantic and each target region and country.[5,6] Using a life table with a radix of one:

$$C_i = \sum_{x=0}^{85+} w_x ({}_n m_{x,i} - {}_n \ddot{m}_{x,i})$$

Component C_i shows the gap in life expectancy attributable to cause i, and is calculated as the difference in the cause-specific mortality rate at age x to $x + n$ between the target location (${}_n m_{x,i}$) and the North Atlantic (${}_n \ddot{m}_{x,i}$), multiplied by a weight w_x representing the contribution of deaths at age x to the overall life expectancy gap, summed across all ages 0–85+.

The weight w_x is calculated as:

$$w_x = n \times \frac{l_x \ddot{e}_x + \ddot{l}_x e_x + l_{x+n} \ddot{e}_{x+n} + \ddot{l}_{x+n} e_{x+n}}{4}$$

where n is the length of the age interval, l_x is the proportion of the original cohort surviving to age x in the target location, and e_x is life expectancy at age x in the target location: with two dots over a letter indicating the same measures for the North Atlantic. For age x = *85*+, the weight is calculated as:

$$w_{85+} = \frac{1}{2} \times \left[\frac{\ddot{T}_{85+}}{M_{85+}} + \frac{T_{85+}}{\ddot{M}_{85+}} \right]$$

© 2025 Karlsson O et al. *JAMA Network Open*.

where T_{85+} is the number of "years" contributed after age *85* and M_{85+} is the all-cause mortality rate at age 85+. The sum of C_i over all causes of death is the total gap in life expectancy between the target location and the North Atlantic.

Uncertainty bounds

The WHO provides uncertainty bounds for number of deaths: We run 1000 Monte Carlo simulations drawing number of deaths using standard errors derived from the uncertainty bounds from the WHO, assuming a lognormal distribution. We constructed 95% uncertainty bounds (UB), defined as percentiles 2.5 and 97.5 from these simulations (our central estimates were calculated using the WHO's central estimate which was close to the median from the simulations in most cases). Note, however, that the WHO acknowledges that their uncertainty estimates do not capture all uncertainty.[3] Further, in some cases, the estimated number of deaths was zero without any uncertainty estimates provided (eg, Malaria in most countries outside sub-Saharan Africa): for these we assign zero deaths in each simulation.

© 2025 Karlsson O et al. *JAMA Network Open*.

eTable 3. Contributions of Causes of Death to Life Expectancy Gaps Relative to the 2019 North Atlantic: All Causes in 2000 and 2019

	China		India		United States		North Atlantic	Central Asia		Central & Eastern Europe		Latin America & Caribbean		Middle East & North Africa		Saharan Africa		Western Pacific & Southeast Asia	
	'00	'19	'00	'19	'00	'19	'00	'00	'19	'00	'19	'00	'19	'00	'19	'00	'19	'00	'19
**Total life expectancy gap**	10	4.32	19.60	11.54	5.45	3.31	3.66	20.75	14.73	13.97	7.61	11.48	7.01	12.87	7.67	31.14	21.61	12.92	7.45
**Total contribution of NCD-7**	7.57	5.48	5.36	6.35	3.95	1.62	2.93	8.53	7.63	9.73	5.91	5.05	2.85	7.61	5.30	4.27	4.96	5.55	4.39
	(76)	(127)	(27)	(55)	(72)	(49)	(80)	(41)	(52)	(70)	(78)	(44)	(41)	(59)	(69)	(14)	(23)	(43)	(59)
Atherosclerotic CVDs	1.32	1.88	1.78	2.47	2.36	0.72	1.89	4.50	4.29	6.77	4.59	2.24	1.20	4.75	3.58	1.18	1.49	1.67	1.41
	(13)	(43)	(9)	(21)	(43)	(22)	(52)	(22)	(29)	(48)	(60)	(19)	(17)	(37)	(47)	(4)	(7)	(13)	(19)
-Ischemic heart disease	0.49	0.97	1.45	2.13	2.16	0.73	1.43	3.58	3.45	4.73	3.39	1.66	0.92	3.50	2.60	0.64	0.83	0.86	0.80
	(5)	(22)	(7)	(18)	(40)	(22)	(39)	(17)	(23)	(34)	(45)	(14)	(13)	(27)	(34)	(2)	(4)	(7)	(11)
-Ischemic stroke	0.83	0.91	0.33	0.33	0.20	-0.01	0.46	0.92	0.84	2.04	1.20	0.58	0.28	1.25	0.98	0.53	0.66	0.81	0.62
	(8)	(21)	(2)	(3)	(4)	(0)	(13)	(4)	(6)	(15)	(16)	(5)	(4)	(10)	(13)	(2)	(3)	(6)	(8)
Diabetes	0.15	0.13	0.32	0.59	0.22	0.19	0.07	0.50	0.70	0.02	0.11	0.85	0.84	0.46	0.55	0.55	0.80	0.40	0.48
	(1)	(3)	(2)	(5)	(4)	(6)	(2)	(2)	(5)	(0)	(2)	(7)	(12)	(4)	(7)	(2)	(4)	(3)	(6)
-Diabetes mellitus	0.04	0.03	0.26	0.48	0.18	0.07	0.07	0.41	0.58	0.03	0.12	0.66	0.64	0.35	0.42	0.45	0.67	0.28	0.32
	(0)	(1)	(1)	(4)	(3)	(2)	(2)	(2)	(4)	(0)	(2)	(6)	(9)	(3)	(6)	(1)	(3)	(2)	(4)
-Chronic kidney disease due to diabetes	0.11	0.11	0.06	0.10	0.04	0.13	-0.01	0.09	0.12	-0.01	-0.01	0.19	0.20	0.11	0.13	0.09	0.13	0.12	0.16
	(1)	(2)	(0)	(1)	(1)	(4)	(0)	(0)	(1)	(0)	(0)	(2)	(3)	(1)	(2)	(0)	(1)	(1)	(2)
Tobacco-related NCDs	2.26	1.20	1.25	1.56	0.77	0.27	0.35	0.78	0.53	0.51	0.08	0.34	-0.07	0.29	0.05	-0.06	-0.08	0.49	0.29
	(23)	(28)	(6)	(14)	(14)	(8)	(10)	(4)	(4)	(4)	(1)	(3)	(-1)	(2)	(1)	(0)	(0)	(4)	(4)
-Chronic obstructive pulmonary disease	2.08	0.95	1.42	1.74	0.37	0.31	0.16	0.82	0.68	0.20	-0.05	0.45	0.19	0.21	0.11	0.19	0.20	0.46	0.33
	(21)	(22)	(7)	(15)	(7)	(9)	(4)	(4)	(5)	(1)	(-1)	(4)	(3)	(2)	(1)	(1)	(1)	(4)	(4)
-Larynx cancer	0	0	0.03	0.03	0.01	0	0.02	0.03	0.02	0.05	0.03	0.03	0.02	0.04	0.03	0	0	0.01	0
	(0)	(0)	(0)	(0)	(0)	(0)	(1)	(0)	(0)	(0)	(0)	(0)	(0)	(0)	(0)	(0)	(0)	(0)	(0)
-Mouth & oropharynx cancers	0.03	0	0.13	0.16	-0.02	-0.02	0.03	0.10	0.10	0.06	0.06	0.01	0	0	-0.02	0.02	0.03	0.08	0.08
	(0)	(0)	(1)	(1)	(0)	(-1)	(1)	(0)	(1)	(0)	(1)	(0)	(0)	(0)	(0)	(0)	(0)	(1)	(1)
-Stomach cancer	0.59	0.38	0.05	0.03	-0.02	-0.06	0.10	0.16	0.08	0.26	0.12	0.22	0.10	0.21	0.13	0.03	0.03	0.24	0.09
	(6)	(9)	(0)	(0)	(0)	(-2)	(3)	(1)	(1)	(2)	(2)	(2)	(1)	(2)	(2)	(0)	(0)	(2)	(1)
-Trachea, bronchus, lung cancers	0.15	0.26	-0.33	-0.38	0.41	-0.02	0.14	-0.17	-0.27	0.20	0.04	-0.15	-0.28	0.04	-0.07	-0.27	-0.31	-0.06	-0.12
	(2)	(6)	(-2)	(-3)	(8)	(0)	(4)	(-1)	(-2)	(1)	(0)	(-1)	(-4)	(0)	(-1)	(-1)	(-1)	(0)	(-2)
Hemorrhagic stroke	2.03	1.31	0.68	0.69	0.16	0.05	0.18	1.23	0.93	0.84	0.39	0.69	0.32	0.69	0.31	1.03	1.06	1.38	1.19
	(20)	(30)	(3)	(6)	(3)	(2)	(5)	(6)	(6)	(6)	(5)	(6)	(5)	(5)	(4)	(3)	(5)	(11)	(16)
Road injury	0.55	0.35	0.38	0.33	0.36	0.24	0.23	0.31	0.31	0.46	0.20	0.46	0.35	0.57	0.37	0.74	0.76	0.56	0.39
	(6)	(8)	(2)	(3)	(7)	(7)	(6)	(1)	(2)	(3)	(3)	(4)	(5)	(4)	(5)	(2)	(4)	(4)	(5)
Suicide	0.15	-0.06	0.24	0.17	0.05	0.16	0.05	0.08	-0.01	0.62	0.21	-0.04	-0.04	-0.09	-0.16	0.07	0.12	0.06	-0.01
	(2)	(-1)	(1)	(1)	(1)	(5)	(1)	(0)	(0)	(4)	(3)	(0)	(-1)	(-1)	(-2)	(0)	(1)	(0)	(0)
Infection-related NCDs	1.10	0.67	0.70	0.55	0.02	-0.01	0.16	1.13	0.88	0.52	0.32	0.51	0.25	0.94	0.59	0.77	0.81	0.99	0.65
	(11)	(15)	(4)	(5)	(0)	(0)	(4)	(5)	(6)	(4)	(4)	(4)	(4)	(7)	(8)	(2)	(4)	(8)	(9)
-Cervix uteri cancer	0.03	0.03	0.11	0.09	0.02	0.01	0.01	0.06	0.05	0.09	0.06	0.16	0.10	0.02	0.01	0.27	0.30	0.10	0.07
	(0)	(1)	(1)	(1)	(0)	(0)	(0)	(0)	(0)	(1)	(1)	(1)	(1)	(0)	(0)	(1)	(1)	(1)	(1)
-Cirrhosis due to hepatitis B	0.20	0.11	0.08	0.08	-0.01	0	0.01	0.15	0.13	0.05	0.05	0.02	0.01	0.09	0.05	0.24	0.27	0.19	0.14
	(2)	(3)	(0)	(1)	(0)	(0)	(0)	(1)	(1)	(0)	(1)	(0)	(0)	(1)	(1)	(1)	(1)	(1)	(2)
-Cirrhosis due to hepatitis C	-0.01	-0.02	0.05	0.05	0.05	0.05	0.03	0.35	0.31	0.07	0.09	0.09	0.04	0.41	0.25	0.08	0.09	0.18	0.15
	(0)	(-1)	(0)	(0)	(1)	(2)	(1)	(2)	(2)	(1)	(1)	(1)	(1)	(3)	(3)	(0)	(0)	(1)	(2)
-Liver cancer secondary to hepatitis B	0.15	0.13	0	0	-0.01	-0.01	0	0.02	0.01	0.01	0.01	0	-0.01	0.04	0.03	0.09	0.08	0.14	0.11
	(1)	(3)	(0)	(0)	(0)	(0)	(0)	(0)	(0)	(0)	(0)	(0)	(0)	(0)	(0)	(0)	(0)	(1)	(2)
-Liver cancer secondary to hepatitis C	-0.02	-0.02	-0.02	-0.02	0	0.01	0	0.02	0.03	-0.01	-0.02	0.01	0.01	0.12	0.10	0	0	0.05	0.02

© 2025 Karlsson O et al. *JAMA Network Open*.

	China		India		United States		North Atlantic	Central Asia		Central & Eastern Europe		Latin America & Caribbean		Middle East & North Africa		Saharan Africa		Western Pacific & Southeast Asia	
	'00	'19	'00	'19	'00	'19	'00	'00	'19	'00	'19	'00	'19	'00	'19	'00	'19	'00	'19
	(0)	(-1)	(0)	(0)	(0)	(0)	(0)	(0)	(0)	(0)	(0)	(0)	(0)	(1)	(1)	(0)	(0)	(0)	(0)
-Rheumatic heart disease	0.17	0.07	0.43	0.32	0	-0.01	0.01	0.36	0.26	0.06	0	0.02	-0.01	0.06	0.02	0.07	0.05	0.09	0.05
	(2)	(2)	(2)	(3)	(0)	(0)	(0)	(2)	(2)	(0)	(0)	(0)	(0)	(0)	(0)	(0)	(0)	(1)	(1)
**Total contribution of I-8**	1.99	0.22	11.87	4.05	0.37	0.05	0.27	8.76	4.53	1.16	0.52	2.68	1.65	2.73	1.16	21.42	11.40	5.86	2.54
	(20)	(5)	(61)	(35)	(7)	(1)	(7)	(42)	(31)	(8)	(7)	(23)	(24)	(21)	(15)	(69)	(53)	(45)	(34)
Childhood-cluster diseases	0.09	0.01	0.83	0.13	0	0	0	1.13	0.28	0.01	0	0.03	0.01	0.17	0.05	1.51	0.45	0.35	0.07
	(1)	(0)	(4)	(1)	(0)	(0)	(0)	(5)	(2)	(0)	(0)	(0)	(0)	(1)	(1)	(5)	(2)	(3)	(1)
Diarrheal diseases	0.16	0	3.16	1.17	-0.01	0.01	-0.01	1.53	0.58	0.02	-0.01	0.30	0.09	0.33	0.05	2.66	1.47	0.86	0.31
	(2)	(0)	(16)	(10)	(0)	(0)	(0)	(7)	(4)	(0)	(0)	(3)	(1)	(3)	(1)	(9)	(7)	(7)	(4)
HIV/AIDS	0.05	0.05	0.59	0.08	0.13	0.03	0.04	0	0.12	0.14	0.27	0.34	0.17	0.01	0.02	5.27	1.67	0.25	0.17
	(0)	(1)	(3)	(1)	(2)	(1)	(1)	(0)	(1)	(1)	(4)	(3)	(2)	(0)	(0)	(17)	(8)	(2)	(2)
Lower respiratory infections	0.60	0.03	1.51	0.78	0.10	-0.07	0.19	1.42	0.69	0.33	0.14	0.82	0.81	0.73	0.37	2.77	2.18	1.19	0.69
	(6)	(1)	(8)	(7)	(2)	(-2)	(5)	(7)	(5)	(2)	(2)	(7)	(12)	(6)	(5)	(9)	(10)	(9)	(9)
Malaria	0	0	0.08	0.02	0	0	0	0.02	0.01	0	0	0.01	0	0.02	0.01	2.31	1.32	0.04	0.01
	(0)	(0)	(0)	(0)	(0)	(0)	(0)	(0)	(0)	(0)	(0)	(0)	(0)	(0)	(0)	(7)	(6)	(0)	(0)
Maternal conditions	0.02	0	0.28	0.05	0	0.01	0	0.41	0.21	0.01	0	0.06	0.04	0.07	0.02	0.96	0.61	0.15	0.06
	(0)	(0)	(1)	(0)	(0)	(0)	(0)	(2)	(1)	(0)	(0)	(0)	(1)	(1)	(0)	(3)	(3)	(1)	(1)
Neonatal conditions	0.87	0.08	2.77	1.16	0.14	0.08	0.04	3.19	2.09	0.32	0.02	0.89	0.43	1.24	0.56	2.13	1.60	1.43	0.65
	(9)	(2)	(14)	(10)	(3)	(2)	(1)	(15)	(14)	(2)	(0)	(8)	(6)	(10)	(7)	(7)	(7)	(11)	(9)
Tuberculosis	0.20	0.04	2.65	0.66	0	0	0.01	1.07	0.55	0.32	0.11	0.23	0.09	0.16	0.07	3.81	2.11	1.58	0.58
	(2)	(1)	(14)	(6)	(0)	(0)	(0)	(5)	(4)	(2)	(1)	(2)	(1)	(1)	(1)	(12)	(10)	(12)	(8)
**Total contribution of other causes**	0.44	-1.38	2.37	1.14	1.13	1.64	0.47	3.46	2.57	3.08	1.18	3.75	2.51	2.53	1.21	5.45	5.25	1.51	0.53
	(4)	(-32)	(12)	(10)	(21)	(50)	(13)	(17)	(17)	(22)	(15)	(33)	(36)	(20)	(16)	(18)	(24)	(12)	(7)
Cardiovascular diseases ex. NCD-7	-0.05	-0.20	-0.03	0.01	0.18	0.17	0.10	0.29	0.35	0.57	0.51	0.33	0.17	0.62	0.42	0.53	0.64	-0.01	-0.06
	(0)	(-5)	(0)	(0)	(3)	(5)	(3)	(1)	(2)	(4)	(7)	(3)	(2)	(5)	(6)	(2)	(3)	(0)	(-1)
Congenital anomalies	0.30	0.05	0.17	0.18	0.09	0.05	0.06	0.25	0.37	0.22	0.05	0.31	0.21	0.39	0.26	0.14	0.22	0.21	0.22
	(3)	(1)	(1)	(2)	(2)	(1)	(2)	(1)	(2)	(2)	(1)	(3)	(3)	(3)	(3)	(0)	(1)	(2)	(3)
Digestive diseases ex. NCD-7	-0.04	-0.20	0.65	0.39	0.05	0.04	0.13	0.41	0.24	0.35	0.43	0.65	0.48	0.08	-0.09	0.49	0.58	0.30	0.17
	(0)	(-5)	(3)	(3)	(1)	(1)	(4)	(2)	(2)	(2)	(6)	(6)	(7)	(1)	(-1)	(2)	(3)	(2)	(2)
Endocrine, blood, immune disorders	-0.09	-0.11	-0.08	-0.10	0.06	0.12	-0.02	0	-0.02	-0.05	-0.07	0.07	0.07	-0.01	-0.04	0.09	0.13	-0.02	-0.03
	(-1)	(-3)	(0)	(-1)	(1)	(4)	(0)	(0)	(0)	(0)	(-1)	(1)	(1)	(0)	(0)	(0)	(1)	(0)	(0)
Genitourinary diseases ex. NCD-7	-0.02	-0.06	0.15	0.21	0.12	0.12	-0.01	0.30	0.36	0.04	0.04	0.29	0.41	0.35	0.42	0.38	0.56	0.19	0.23
	(0)	(-1)	(1)	(2)	(2)	(4)	(0)	(1)	(2)	(0)	(1)	(3)	(6)	(3)	(5)	(1)	(3)	(1)	(3)
Infectious & parasitic diseases ex. I-8	0.11	-0.06	0.67	0.27	0.14	0.11	0	0.82	0.37	0.02	-0.05	0.34	0.21	0.17	0	1.58	0.99	0.35	0.15
	(1)	(-1)	(3)	(2)	(3)	(3)	(0)	(4)	(3)	(0)	(-1)	(3)	(3)	(1)	(0)	(5)	(5)	(3)	(2)
Violence	0.06	0	0.13	0.08	0.19	0.19	0.02	0.28	0.43	0.42	0.10	0.91	0.90	0.21	0.45	0.86	0.54	0.18	0.12
	(1)	(0)	(1)	(1)	(3)	(6)	(0)	(1)	(3)	(3)	(1)	(8)	(13)	(2)	(6)	(3)	(2)	(1)	(2)
Cancers ex. NCD-7	-0.19	-0.49	-0.68	-0.79	0.29	-0.23	0.46	-0.08	-0.21	0.38	0.18	0.19	-0.10	0.27	0.04	0.13	0.45	-0.17	-0.32
	(-2)	(-11)	(-3)	(-7)	(5)	(-7)	(12)	(0)	(-1)	(3)	(2)	(2)	(-1)	(2)	(1)	(0)	(2)	(-1)	(-4)
Mental & substance use disorders	-0.08	-0.12	-0.09	-0.09	0.04	0.53	-0.02	-0.04	-0.09	0.40	0.15	-0.03	-0.08	-0.10	-0.12	-0.07	-0.07	-0.10	-0.11
	(-1)	(-3)	(0)	(-1)	(1)	(16)	(-1)	(0)	(-1)	(3)	(2)	(0)	(-1)	(-1)	(-2)	(0)	(0)	(-1)	(-2)
Musculoskeletal diseases	-0.03	-0.03	-0.01	0	0.03	0	0	0	0	-0.02	-0.03	0.02	0.02	-0.02	-0.03	-0.02	-0.02	-0.01	-0.01
	(0)	(-1)	(0)	(0)	(0)	(0)	(0)	(0)	(0)	(0)	(0)	(0)	(0)	(0)	(0)	(0)	(0)	(0)	(0)
Neurological conditions	-0.21	-0.22	-0.22	-0.27	-0.20	0.44	-0.37	-0.05	-0.08	-0.28	-0.34	-0.38	-0.34	-0.06	-0.20	-0.09	-0.01	-0.42	-0.37
	(-2)	(-5)	(-1)	(-2)	(-4)	(13)	(-10)	(0)	(-1)	(-2)	(-4)	(-3)	(-5)	(-1)	(-3)	(0)	(0)	(-3)	(-5)
Nutritional deficiencies	0.03	0	0.33	0.05	0.01	0.03	0	0.20	0.07	0	-0.01	0.30	0.11	0.06	0.01	0.50	0.23	0.27	0.07
	(0)	(0)	(2)	(0)	(0)	(1)	(0)	(1)	(0)	(0)	(0)	(3)	(2)	(0)	(0)	(2)	(1)	(2)	(1)

© 2025 Karlsson O et al. *JAMA Network Open*.

	China		India		United States		North Atlantic	Central Asia		Central & Eastern Europe		Latin America & Caribbean		Middle East & North Africa		Saharan Africa		Western Pacific & Southeast Asia	
	'00	'19	'00	'19	'00	'19	'00	'00	'19	'00	'19	'00	'19	'00	'19	'00	'19	'00	'19
Other neoplasms	-0.07	-0.08	-0.06	-0.07	-0.01	-0.03	0.01	-0.05	-0.06	-0.05	-0.03	-0.01	0.01	-0.06	-0.07	-0.05	-0.06	-0.04	-0.05
	(-1)	(-2)	(0)	(-1)	(0)	(-1)	(0)	(0)	(0)	(0)	(0)	(0)	(0)	(0)	(-1)	(0)	(0)	(0)	(-1)
Respiratory diseases ex. NCD-7	-0.03	-0.09	0.42	0.41	0.04	0.01	0.05	0.29	0.17	-0.01	-0.09	0.24	0.11	0.15	0.02	0.19	0.18	0.22	0.14
	(0)	(-2)	(2)	(4)	(1)	(0)	(1)	(1)	(1)	(0)	(-1)	(2)	(2)	(1)	(0)	(1)	(1)	(2)	(2)
Respiratory infections ex. I-8	0.03	0	0	0	0	0	0	0.02	0	0.01	0	0.01	0	0	0	0.04	0.04	0	0
	(0)	(0)	(0)	(0)	(0)	(0)	(0)	(0)	(0)	(0)	(0)	(0)	(0)	(0)	(0)	(0)	(0)	(0)	(0)
Skin diseases	-0.02	-0.02	0	-0.01	0	0	0	0	0	0	0.01	0.03	0.06	0	0	0.02	0.03	0.01	0.03
	(0)	(0)	(0)	(0)	(0)	(0)	(0)	(0)	(0)	(0)	(0)	(0)	(1)	(0)	(0)	(0)	(0)	(0)	(0)
Sudden infant death syndrome	0	0	0.05	0.01	0.03	0.02	0.01	0.06	0.01	0.02	0	0.01	0	0.03	0.01	0.03	0.01	0.02	0.01
	(0)	(0)	(0)	(0)	(1)	(1)	(0)	(0)	(0)	(0)	(0)	(0)	(0)	(0)	(0)	(0)	(0)	(0)	(0)
Unintentional injuries ex. NCD-7	0.73	0.26	0.97	0.87	0.08	0.09	0.05	0.76	0.64	1.05	0.33	0.46	0.24	0.46	0.13	0.68	0.82	0.52	0.35
	(7)	(6)	(5)	(8)	(1)	(3)	(1)	(4)	(4)	(8)	(4)	(4)	(3)	(4)	(2)	(2)	(4)	(4)	(5)

Note: Number of years are shown (percentage of the total gap in parentheses below). Both 2000 and 2019 were compared to the North Atlantic in 2019 (which had a life expectancy of 82 years). Negative contribution suggests that the gap in life expectancy would have been greater had it not been for lower mortality from that cause than in the 2019 North Atlantic. Therefore, the contribution of some causes or groups of causes can be greater than the total life expectancy gap. I-8 = 8 priority infectious and maternal and child health conditions. NCD-7 = 7 priority noncommunicable diseases (NCD) and injuries. ex. = excluding. CVD = cardiovascular diseases.

© 2025 Karlsson O et al. *JAMA Network Open*.

eTable 4. Contributions of Causes of Death to Life Expectancy Gaps Relative to the 2019 North Atlantic: Results for the Year 2000

	China	India	United States	North Atlantic	Central Asia	Central & Eastern Europe	Latin America & Caribbean	Middle East & North Africa	Sub-Saharan Africa	Western Pacific & Southeast Asia
**Total life expectancy gap**	10	19.60	5.45	3.66	20.75	13.97	11.48	12.87	31.14	12.92
**Total contribution of NCD-7**	7.57 (76)	5.36 (27)	3.95 (72)	2.93 (80)	8.53 (41)	9.73 (70)	5.05 (44)	7.61 (59)	4.27 (14)	5.55 (43)
	[7.18, 7.93]	[4.92, 5.74]	[3.71, 4.16]	[2.82, 3.04]	[8.08, 8.83]	[9.61, 9.83]	[4.96, 5.13]	[7.28, 7.81]	[4.09, 4.34]	[5.34, 5.67]
Atherosclerotic CVDs	1.32 (13)	1.78 (9)	2.36 (43)	1.89 (52)	4.50 (22)	6.77 (48)	2.24 (19)	4.75 (37)	1.18 (4)	1.67 (13)
	[1.20, 1.44]	[1.59, 1.93]	[2.16, 2.53]	[1.81, 1.97]	[4.21, 4.68]	[6.67, 6.84]	[2.18, 2.28]	[4.51, 4.86]	[1.10, 1.22]	[1.56, 1.73]
Diabetes	0.15 (1)	0.32 (2)	0.22 (4)	0.07 (2)	0.50 (2)	0.02 (0)	0.85 (7)	0.46 (4)	0.55 (2)	0.40 (3)
	[0.13, 0.17]	[0.29, 0.35]	[0.21, 0.24]	[0.06, 0.08]	[0.46, 0.55]	[0.02, 0.03]	[0.83, 0.87]	[0.43, 0.49]	[0.52, 0.56]	[0.38, 0.41]
Infection-related NCDs	1.10 (11)	0.70 (4)	0.02 (0)	0.16 (4)	1.13 (5)	0.52 (4)	0.51 (4)	0.94 (7)	0.77 (2)	0.99 (8)
	[1.05, 1.15]	[0.64, 0.78]	[0.01, 0.02]	[0.15, 0.17]	[1.07, 1.22]	[0.52, 0.53]	[0.50, 0.52]	[0.89, 1.01]	[0.76, 0.80]	[0.97, 1.02]
Tobacco-related NCDs	2.26 (23)	1.25 (6)	0.77 (14)	0.35 (10)	0.78 (4)	0.51 (4)	0.34 (3)	0.29 (2)	-0.06 (0)	0.49 (4)
	[2.11, 2.42]	[1.07, 1.43]	[0.73, 0.82]	[0.33, 0.38]	[0.67, 0.88]	[0.50, 0.53]	[0.32, 0.37]	[0.25, 0.34]	[-0.08, -0.04]	[0.45, 0.53]
Hemorrhagic stroke	2.03 (20)	0.68 (3)	0.16 (3)	0.18 (5)	1.23 (6)	0.84 (6)	0.69 (6)	0.69 (5)	1.03 (3)	1.38 (11)
	[1.89, 2.16]	[0.60, 0.77]	[0.15, 0.17]	[0.17, 0.19]	[1.14, 1.32]	[0.82, 0.85]	[0.67, 0.70]	[0.65, 0.74]	[0.97, 1.06]	[1.32, 1.43]
Road injury	0.55 (6)	0.38 (2)	0.36 (7)	0.23 (6)	0.31 (1)	0.46 (3)	0.46 (4)	0.57 (4)	0.74 (2)	0.56 (4)
	[0.53, 0.58]	[0.36, 0.41]	[0.36, 0.37]	[0.23, 0.23]	[0.29, 0.32]	[0.45, 0.46]	[0.45, 0.46]	[0.54, 0.57]	[0.69, 0.74]	[0.54, 0.57]
Suicide	0.15 (2)	0.24 (1)	0.05 (1)	0.05 (1)	0.08 (0)	0.62 (4)	-0.04 (0)	-0.09 (-1)	0.07 (0)	0.06 (0)
	[0.14, 0.17]	[0.21, 0.27]	[0.05, 0.06]	[0.05, 0.05]	[0.06, 0.11]	[0.61, 0.62]	[-0.04, -0.04]	[-0.10, -0.09]	[0.06, 0.08]	[0.05, 0.06]
**Total contribution of I-8**	1.99 (20)	11.87 (61)	0.37 (7)	0.27 (7)	8.76 (42)	1.16 (8)	2.68 (23)	2.73 (21)	21.42 (69)	5.86 (45)
	[1.81, 2.23]	[10.98, 12.98]	[0.34, 0.40]	[0.25, 0.28]	[7.96, 9.74]	[1.13, 1.19]	[2.63, 2.74]	[2.57, 2.85]	[20.57, 22.19]	[5.63, 6.09]
Childhood-cluster diseases	0.09 (1)	0.83 (4)	0 (0)	0 (0)	1.13 (5)	0.01 (0)	0.03 (0)	0.17 (1)	1.51 (5)	0.35 (3)
	[0.06, 0.16]	[0.49, 1.52]	[0, 0]	[0, 0]	[0.84, 1.81]	[0.01, 0.01]	[0.02, 0.05]	[0.15, 0.22]	[1.38, 1.95]	[0.31, 0.46]
Diarrheal diseases	0.16 (2)	3.16 (16)	-0.01 (0)	-0.01 (0)	1.53 (7)	0.02 (0)	0.30 (3)	0.33 (3)	2.66 (9)	0.86 (7)
	[0.13, 0.19]	[2.76, 3.76]	[-0.01, 0]	[-0.02, -0.01]	[1.32, 1.84]	[0.02, 0.03]	[0.29, 0.31]	[0.30, 0.35]	[2.52, 2.81]	[0.80, 0.94]
HIV/AIDS	0.05 (0)	0.59 (3)	0.13 (2)	0.04 (1)	0 (0)	0.14 (1)	0.34 (3)	0.01 (0)	5.27 (17)	0.25 (2)
	[0.04, 0.05]	[0.52, 0.69]	[0.13, 0.13]	[0.04, 0.04]	[0, 0]	[0.14, 0.14]	[0.33, 0.35]	[0.01, 0.01]	[5.04, 5.40]	[0.23, 0.25]
Lower respiratory infections	0.60 (6)	1.51 (8)	0.10 (2)	0.19 (5)	1.42 (7)	0.33 (2)	0.82 (7)	0.73 (6)	2.77 (9)	1.19 (9)
	[0.47, 0.78]	[1.31, 1.72]	[0.07, 0.12]	[0.18, 0.21]	[1.20, 1.64]	[0.32, 0.35]	[0.79, 0.85]	[0.66, 0.78]	[2.58, 2.85]	[1.09, 1.29]
Malaria	0 (0)	0.08 (0)	0 (0)	0 (0)	0.02 (0)	0 (0)	0.01 (0)	0.02 (0)	2.31 (7)	0.04 (0)
	[0, 0]	[0.07, 0.10]	[na]	[na]	[0.02, 0.03]	[na]	[0.01, 0.01]	[0.02, 0.05]	[2.02, 2.66]	[0.04, 0.05]
Maternal conditions	0.02 (0)	0.28 (1)	0 (0)	0 (0)	0.41 (2)	0.01 (0)	0.06 (0)	0.07 (1)	0.96 (3)	0.15 (1)
	[0.02, 0.02]	[0.25, 0.31]	[0, 0]	[0, 0]	[0.36, 0.46]	[0.01, 0.01]	[0.05, 0.06]	[0.06, 0.07]	[0.91, 0.99]	[0.14, 0.16]
Neonatal conditions	0.87 (9)	2.77 (14)	0.14 (3)	0.04 (1)	3.19 (15)	0.32 (2)	0.89 (8)	1.24 (10)	2.13 (7)	1.43 (11)
	[0.73, 1.00]	[2.32, 3.14]	[0.12, 0.16]	[0.03, 0.05]	[2.69, 3.60]	[0.30, 0.34]	[0.84, 0.94]	[1.10, 1.34]	[1.88, 2.33]	[1.26, 1.57]
Tuberculosis	0.20 (2)	2.65 (14)	0 (0)	0.01 (0)	1.07 (5)	0.32 (2)	0.23 (2)	0.16 (1)	3.81 (12)	1.58 (12)
	[0.19, 0.22]	[2.46, 2.84]	[0, 0]	[0.01, 0.01]	[1.01, 1.11]	[0.32, 0.33]	[0.23, 0.24]	[0.15, 0.17]	[3.67, 3.92]	[1.52, 1.64]
**Total contribution of other causes**	0.44 (4)	2.37 (12)	1.13 (21)	0.47 (13)	3.46 (17)	3.08 (22)	3.75 (33)	2.53 (20)	5.45 (18)	1.51 (12)
	[-0.01, 0.89]	[1.14, 3.45]	[0.90, 1.39]	[0.34, 0.58]	[2.42, 4.43]	[2.98, 3.20]	[3.65, 3.86]	[2.31, 2.94]	[4.67, 6.35]	[1.23, 1.85]

© 2025 Karlsson O et al. *JAMA Network Open*.

Note: Number of years are shown (percentage of the total gap in parentheses) with 95% uncertainty bounds in square brackets below. Comparisons were made to the 2019 North Atlantic (which had a life expectancy of 82 years). Negative contribution suggests that the gap in life expectancy would have been greater had it not been for lower mortality from that cause than in the 2019 North Atlantic. Therefore, the contribution of some causes or groups of causes can be over 100% of the total life expectancy gap. I-8 = 8 priority infectious and maternal and child health conditions. NCD-7 = 7 priority noncommunicable diseases (NCD) and injuries. CVD = cardiovascular diseases.

© 2025 Karlsson O et al. *JAMA Network Open*.

eTable 5. Contributions of Causes of Death to Life Expectancy Gaps Relative to the 2019 North Atlantic: Results for the Year 2021

	China	India	United States	North Atlantic	Central Asia	Central & Eastern Europe	Latin America & Caribbean	Middle East & North Africa	Sub-Saharan Africa	Western Pacific & Southeast Asia
**Total life expectancy gap**	4.14	15.04	5.86	0.38	16.01	10.46	10.44	9.50	22.04	8.88
**Total contribution of NCD-7**	5.37 (130)	4.92 (33)	1.73 (30)	-0.23 (-62)	6.55 (41)	4.65 (44)	2.47 (24)	4.23 (45)	4.61 (21)	3.78 (43)
	[4.80, 5.93]	[4.63, 5.17]	[1.48, 1.96]	[-0.35, -0.11]	[6.13, 6.87]	[4.46, 4.81]	[2.38, 2.56]	[4.01, 4.37]	[4.44, 4.67]	[3.48, 3.90]
Atherosclerotic CVDs	1.85 (45)	1.90 (13)	0.76 (13)	-0.11 (-30)	3.70 (23)	3.63 (35)	1.14 (11)	2.89 (30)	1.40 (6)	1.20 (14)
	[1.56, 2.15]	[1.77, 2.04]	[0.59, 0.92]	[-0.18, -0.03]	[3.41, 3.90]	[3.47, 3.76]	[1.09, 1.20]	[2.72, 2.99]	[1.31, 1.44]	[1.06, 1.27]
Diabetes	0.13 (3)	0.47 (3)	0.24 (4)	0 (1)	0.64 (4)	0.11 (1)	0.79 (8)	0.47 (5)	0.78 (4)	0.44 (5)
	[0.11, 0.17]	[0.43, 0.51]	[0.22, 0.27]	[-0.01, 0.01]	[0.58, 0.71]	[0.10, 0.12]	[0.77, 0.82]	[0.44, 0.50]	[0.75, 0.80]	[0.41, 0.46]
Infection-related NCDs	0.64 (16)	0.48 (3)	0.02 (0)	-0.01 (-2)	0.76 (5)	0.27 (3)	0.19 (2)	0.45 (5)	0.70 (3)	0.57 (6)
	[0.58, 0.71]	[0.44, 0.54]	[0.01, 0.03]	[-0.02, 0]	[0.71, 0.82]	[0.26, 0.29]	[0.19, 0.20]	[0.42, 0.49]	[0.68, 0.72]	[0.54, 0.60]
Tobacco-related NCDs	1.19 (29)	1.15 (8)	0.14 (2)	-0.08 (-22)	0.38 (2)	0.01 (0)	-0.22 (-2)	-0.03 (0)	-0.12 (-1)	0.21 (2)
	[1.00, 1.41]	[1.02, 1.27]	[0.08, 0.19]	[-0.11, -0.05]	[0.29, 0.48]	[-0.01, 0.04]	[-0.24, -0.20]	[-0.07, 0.01]	[-0.14, -0.09]	[0.14, 0.24]
Hemorrhagic stroke	1.28 (31)	0.55 (4)	0.07 (1)	-0.01 (-3)	0.81 (5)	0.32 (3)	0.27 (3)	0.25 (3)	1.02 (5)	1.04 (12)
	[1.14, 1.43]	[0.49, 0.61]	[0.06, 0.09]	[-0.02, 0]	[0.73, 0.90]	[0.31, 0.34]	[0.26, 0.28]	[0.22, 0.27]	[0.98, 1.05]	[0.96, 1.09]
Road injury	0.33 (8)	0.26 (2)	0.30 (5)	-0.01 (-3)	0.28 (2)	0.14 (1)	0.32 (3)	0.36 (4)	0.71 (3)	0.33 (4)
	[0.31, 0.35]	[0.24, 0.27]	[0.29, 0.31]	[-0.01, -0.01]	[0.26, 0.29]	[0.13, 0.14]	[0.31, 0.32]	[0.34, 0.36]	[0.66, 0.70]	[0.31, 0.33]
Suicide	-0.06 (-1)	0.10 (1)	0.19 (3)	-0.01 (-2)	-0.03 (0)	0.16 (2)	-0.03 (0)	-0.16 (-2)	0.12 (1)	-0.01 (0)
	[-0.07, -0.04]	[0.09, 0.12]	[0.18, 0.19]	[-0.01, 0]	[-0.05, -0.01]	[0.15, 0.17]	[-0.04, -0.03]	[-0.16, -0.15]	[0.11, 0.13]	[-0.02, -0.01]
**Total contribution of I-8**	0.15 (4)	3.21 (21)	0.01 (0)	-0.08 (-21)	4.10 (26)	0.51 (5)	1.30 (12)	1.00 (11)	9.99 (45)	2.31 (26)
	[0.10, 0.21]	[2.97, 3.51]	[-0.02, 0.04]	[-0.10, -0.06]	[3.77, 4.50]	[0.49, 0.53]	[1.26, 1.35]	[0.91, 1.13]	[9.59, 10.34]	[2.19, 2.42]
Childhood-cluster diseases	0.01 (0)	0.07 (0)	0 (0)	0 (0)	0.18 (1)	0 (0)	0 (0)	0.02 (0)	0.24 (1)	0.03 (0)
	[0, 0.01]	[0.04, 0.14]	[0, 0]	[0, 0]	[0.13, 0.28]	[0, 0]	[0, 0.01]	[0.01, 0.03]	[0.21, 0.36]	[0.03, 0.04]
Diarrheal diseases	0 (0)	0.83 (6)	0.01 (0)	-0.01 (-2)	0.48 (3)	-0.01 (0)	0.07 (1)	0.05 (1)	1.32 (6)	0.27 (3)
	[0, 0.01]	[0.71, 1.04]	[0.01, 0.01]	[-0.01, 0]	[0.40, 0.62]	[-0.01, -0.01]	[0.07, 0.08]	[0.03, 0.10]	[1.25, 1.45]	[0.25, 0.31]
HIV/AIDS	0.05 (1)	0.07 (0)	0.02 (0)	0 (0)	0.14 (1)	0.30 (3)	0.14 (1)	0.02 (0)	1.34 (6)	0.14 (2)
	[0.05, 0.06]	[0.06, 0.09]	[0.02, 0.02]	[0, 0]	[0.11, 0.30]	[0.29, 0.30]	[0.14, 0.14]	[0.02, 0.02]	[1.29, 1.38]	[0.13, 0.16]
Lower respiratory infections	0.01 (0)	0.59 (4)	-0.09 (-2)	-0.07 (-18)	0.56 (3)	0.11 (1)	0.53 (5)	0.28 (3)	1.95 (9)	0.52 (6)
	[-0.02, 0.05]	[0.50, 0.70]	[-0.11, -0.08]	[-0.08, -0.05]	[0.45, 0.71]	[0.10, 0.12]	[0.51, 0.56]	[0.23, 0.40]	[1.81, 2.08]	[0.47, 0.59]
Malaria	0 (0)	0.02 (0)	0 (0)	0 (0)	0 (0)	0 (0)	0 (0)	0.02 (0)	1.31 (6)	0.01 (0)
	[na]	[0.02, 0.03]	[na]	[na]	[0, 0.01]	[na]	[0, 0]	[0.01, 0.02]	[1.16, 1.44]	[0.01, 0.02]
Maternal conditions	0 (0)	0.05 (0)	0.01 (0)	0 (0)	0.21 (1)	0 (0)	0.05 (1)	0.03 (0)	0.59 (3)	0.06 (1)
	[0, 0]	[0.05, 0.05]	[0.01, 0.01]	[0, 0]	[0.18, 0.26]	[0, 0]	[0.05, 0.06]	[0.03, 0.03]	[0.56, 0.65]	[0.06, 0.07]
Neonatal conditions	0.04 (1)	0.99 (7)	0.06 (1)	-0.01 (-2)	1.95 (12)	0 (0)	0.40 (4)	0.53 (6)	1.51 (7)	0.60 (7)
	[0, 0.07]	[0.76, 1.18]	[0.04, 0.09]	[-0.02, 0]	[1.59, 2.18]	[-0.01, 0.01]	[0.36, 0.43]	[0.45, 0.58]	[1.33, 1.65]	[0.51, 0.68]
Tuberculosis	0.04 (1)	0.58 (4)	0 (0)	0 (0)	0.57 (4)	0.10 (1)	0.11 (1)	0.07 (1)	1.72 (8)	0.67 (8)
	[0.04, 0.04]	[0.54, 0.63]	[0, 0]	[0, 0]	[0.53, 0.59]	[0.10, 0.11]	[0.10, 0.11]	[0.06, 0.07]	[1.64, 1.75]	[0.64, 0.68]
**Total contribution of other causes**	-1.37 (-33)	6.92 (46)	4.12 (70)	0.70 (183)	5.36 (33)	5.30 (51)	6.66 (64)	4.27 (45)	7.45 (34)	2.79 (31)
	[-1.95, -0.80]	[6.49, 7.27]	[3.88, 4.37]	[0.56, 0.82]	[4.83, 5.92]	[5.13, 5.49]	[6.56, 6.77]	[4.08, 4.52]	[7.08, 7.92]	[2.60, 3.15]
COVID-19	0 (0)	4.86 (32)	1.88 (32)	0.91 (240)	2.25 (14)	3.56 (34)	4.46 (43)	2.71 (28)	1.92 (9)	1.70 (19)

© 2025 Karlsson O et al. *JAMA Network Open*.

	China	India	United States	North Atlantic	Central Asia	Central & Eastern Europe	Latin America & Caribbean	Middle East & North Africa	Sub-Saharan Africa	Western Pacific & Southeast Asia
	[0, 0]	[4.68, 4.97]	[1.81, 1.92]	[0.88, 0.92]	[2.11, 2.32]	[3.40, 3.73]	[4.32, 4.55]	[2.58, 2.75]	[1.84, 1.90]	[1.53, 1.96]
COVID-19 pandemic-related	0.01 (0)	1.11 (7)	0 (0)	0 (0)	0.83 (5)	0.68 (7)	0.15 (1)	0.88 (9)	0.75 (3)	0.68 (8)
	[0.01, 0.03]	[1.02, 1.19]	[na]	[0, 0]	[0.78, 0.98]	[0.64, 0.76]	[0.14, 0.17]	[0.84, 1.01]	[0.73, 0.78]	[0.60, 1.04]

Note: Number of years are shown (percentage of the total gap in parentheses) with 95% uncertainty bounds in square brackets below. Comparisons were made to the 2019 North Atlantic (which had a life expectancy of 82 years). Negative contribution suggests that the gap in life expectancy would have been greater had it not been for lower mortality from that cause than in the 2019 North Atlantic. Therefore, the contribution of some causes or groups of causes can be over 100% of the total life expectancy gap. I-8 = 8 priority infectious and maternal and child health conditions. NCD-7 = 7 priority noncommunicable diseases (NCD) and injuries. CVD = cardiovascular diseases.

© 2025 Karlsson O et al. *JAMA Network Open*.

eTable 6. Contributions of Causes of Death to Life Expectancy Gaps Relative to the North Atlantic, 2019: All Causes for Males (M) and Females (F)

	China		India		United States		Central Asia		Central & Eastern Europe		Latin America & Caribbean		Middle East & North Africa		Saharan Africa		Western Pacific & Southeast Asia	
	M	F	M	F	M	F	M	F	M	F	M	F	M	F	M	F	M	F
**Total life expectancy gap**	4.63	3.76	10.64	12.26	3.44	3.02	15.12	14.07	9.88	5.41	7.66	6.24	7.52	7.59	21.20	21.93	8.07	6.72
**Total contribution of NCD-7**	5.54	5.13	6.27	6.19	1.64	1.54	7.64	7.54	6.71	5.11	2.71	2.97	5.10	5.31	4.69	5.24	4.61	4.06
	(120)	(136)	(59)	(50)	(48)	(51)	(51)	(54)	(68)	(94)	(35)	(48)	(68)	(70)	(22)	(24)	(57)	(60)
Atherosclerotic CVDs	1.71	1.99	2.62	2.18	0.78	0.62	4.05	4.49	4.60	4.53	1.14	1.25	3.19	3.87	1.31	1.66	1.36	1.44
	(37)	(53)	(25)	(18)	(23)	(21)	(27)	(32)	(47)	(84)	(15)	(20)	(42)	(51)	(6)	(8)	(17)	(21)
-Ischemic heart disease	0.77	1.16	2.31	1.83	0.81	0.61	3.31	3.56	3.52	3.24	0.87	0.96	2.39	2.73	0.70	0.96	0.79	0.80
	(17)	(31)	(22)	(15)	(23)	(20)	(22)	(25)	(36)	(60)	(11)	(15)	(32)	(36)	(3)	(4)	(10)	(12)
-Ischemic stroke	0.94	0.82	0.31	0.35	-0.03	0.01	0.74	0.93	1.09	1.29	0.28	0.28	0.80	1.14	0.61	0.71	0.58	0.64
	(20)	(22)	(3)	(3)	(-1)	(0)	(5)	(7)	(11)	(24)	(4)	(5)	(11)	(15)	(3)	(3)	(7)	(9)
Diabetes	0.10	0.16	0.52	0.65	0.23	0.15	0.54	0.83	0.08	0.16	0.72	0.95	0.43	0.66	0.74	0.86	0.38	0.58
	(2)	(4)	(5)	(5)	(7)	(5)	(4)	(6)	(1)	(3)	(9)	(15)	(6)	(9)	(3)	(4)	(5)	(9)
-Diabetes mellitus	0	0.06	0.40	0.57	0.10	0.03	0.42	0.71	0.08	0.17	0.52	0.75	0.32	0.52	0.60	0.74	0.23	0.41
	(0)	(2)	(4)	(5)	(3)	(1)	(3)	(5)	(1)	(3)	(7)	(12)	(4)	(7)	(3)	(3)	(3)	(6)
-Chronic kidney disease due to diabetes	0.10	0.11	0.12	0.08	0.13	0.12	0.12	0.12	0	-0.01	0.20	0.20	0.11	0.15	0.14	0.13	0.15	0.16
	(2)	(3)	(1)	(1)	(4)	(4)	(1)	(1)	(0)	(0)	(3)	(3)	(1)	(2)	(1)	(1)	(2)	(2)
Tobacco-related NCDs	1.26	1.04	1.42	1.65	0.05	0.50	0.91	0.21	0.57	-0.33	-0.13	0	0.26	-0.16	-0.11	-0.03	0.48	0.08
	(27)	(28)	(13)	(13)	(2)	(17)	(6)	(1)	(6)	(-6)	(-2)	(0)	(3)	(-2)	(-1)	(0)	(6)	(1)
-Chronic obstructive pulmonary disease	0.92	0.91	1.61	1.83	0.22	0.40	0.92	0.44	0.09	-0.16	0.18	0.20	0.15	0.07	0.22	0.19	0.43	0.21
	(20)	(24)	(15)	(15)	(6)	(13)	(6)	(3)	(1)	(-3)	(2)	(3)	(2)	(1)	(1)	(1)	(5)	(3)
-Larynx cancer	0	0	0.05	0.01	-0.01	0	0.04	0.01	0.06	0	0.03	0	0.05	0.01	0.01	0	0.01	0
	(0)	(0)	(0)	(0)	(0)	(0)	(0)	(0)	(1)	(0)	(0)	(0)	(1)	(0)	(0)	(0)	(0)	(0)
-Mouth & oropharynx cancers	0	0	0.20	0.11	-0.03	-0.01	0.14	0.06	0.12	0.01	0	0	-0.03	0	0.02	0.03	0.11	0.04
	(0)	(0)	(2)	(1)	(-1)	(0)	(1)	(0)	(1)	(0)	(0)	(0)	(0)	(0)	(0)	(0)	(1)	(1)
-Stomach cancer	0.49	0.23	0.04	0.03	-0.07	-0.04	0.12	0.05	0.17	0.08	0.12	0.09	0.17	0.09	0.02	0.04	0.12	0.06
	(11)	(6)	(0)	(0)	(-2)	(-1)	(1)	(0)	(2)	(2)	(2)	(1)	(2)	(1)	(0)	(0)	(1)	(1)
-Trachea, bronchus, lung cancers	0.35	0.14	-0.44	-0.30	-0.13	0.11	-0.20	-0.30	0.29	-0.18	-0.34	-0.20	0.10	-0.24	-0.36	-0.25	-0.07	-0.17
	(8)	(4)	(-4)	(-2)	(-4)	(3)	(-1)	(-2)	(3)	(-3)	(-4)	(-3)	(1)	(-3)	(-2)	(-1)	(-1)	(-3)
Hemorrhagic stroke	1.37	1.19	0.67	0.69	0.05	0.05	0.85	0.98	0.46	0.32	0.31	0.34	0.26	0.34	0.94	1.17	1.15	1.21
	(30)	(32)	(6)	(6)	(1)	(2)	(6)	(7)	(5)	(6)	(4)	(5)	(4)	(5)	(4)	(5)	(14)	(18)
Road injury	0.42	0.23	0.47	0.14	0.30	0.16	0.50	0.14	0.28	0.10	0.54	0.14	0.50	0.20	1.03	0.46	0.57	0.16
	(9)	(6)	(4)	(1)	(9)	(5)	(3)	(1)	(3)	(2)	(7)	(2)	(7)	(3)	(5)	(2)	(7)	(2)
Suicide	-0.14	0.05	0.12	0.22	0.25	0.06	0.02	-0.02	0.38	0.03	-0.03	-0.04	-0.22	-0.08	0.19	0.05	-0.03	0.01
	(-3)	(1)	(1)	(2)	(7)	(2)	(0)	(0)	(4)	(0)	(0)	(-1)	(-3)	(-1)	(1)	(0)	(0)	(0)
Infection-related NCDs	0.82	0.47	0.45	0.67	-0.02	0	0.78	0.93	0.34	0.31	0.18	0.33	0.68	0.48	0.59	1.05	0.69	0.58
	(18)	(12)	(4)	(5)	(-1)	(0)	(5)	(7)	(3)	(6)	(2)	(5)	(9)	(6)	(3)	(5)	(9)	(9)
-Cervix uteri cancer	0	0.07	0	0.19	0	0.01	0	0.08	0	0.13	0	0.20	0	0.02	0	0.64	0	0.16
	(0)	(2)	(0)	(2)	(0)	(0)	(0)	(1)	(0)	(2)	(0)	(3)	(0)	(0)	(0)	(3)	(0)	(2)

© 2025 Karlsson O et al. *JAMA Network Open*.

	China		India		United States		Central Asia		Central & Eastern Europe		Latin America & Caribbean		Middle East & North Africa		Saharan Africa		Western Pacific & Southeast Asia	
	M	F	M	F	M	F	M	F	M	F	M	F	M	F	M	F	M	F
-Cirrhosis due to hepatitis B	0.15	0.06	0.12	0.04	-0.01	0	0.14	0.12	0.06	0.04	0.01	0	0.06	0.04	0.33	0.19	0.17	0.10
	(3)	(2)	(1)	(0)	(0)	(0)	(1)	(1)	(1)	(1)	(0)	(0)	(1)	(1)	(2)	(1)	(2)	(2)
-Cirrhosis due to hepatitis C	-0.03	-0.01	0.05	0.04	0.06	0.04	0.25	0.35	0.11	0.07	0.06	0.02	0.27	0.21	0.10	0.07	0.16	0.12
	(-1)	(0)	(1)	(0)	(2)	(1)	(2)	(3)	(1)	(1)	(1)	(0)	(4)	(3)	(0)	(0)	(2)	(2)
-Liver cancer secondary to hepatitis B	0.19	0.05	0	0	-0.01	-0.01	0.02	0.01	0.01	0	-0.01	0	0.04	0.02	0.12	0.05	0.17	0.05
	(4)	(1)	(0)	(0)	(0)	(0)	(0)	(0)	(0)	(0)	(0)	(0)	(1)	(0)	(1)	(0)	(2)	(1)
-Liver cancer secondary to hepatitis C	-0.03	-0.02	-0.03	-0.02	0.02	0.01	0.03	0.03	-0.02	-0.01	0	0.02	0.12	0.07	-0.01	0	0.03	0.02
	(-1)	(0)	(0)	(0)	(0)	(0)	(0)	(0)	(0)	(0)	(0)	(0)	(2)	(1)	(0)	(0)	(0)	(0)
-Rheumatic heart disease	0.05	0.08	0.26	0.39	-0.01	-0.01	0.22	0.29	0	0	0	-0.01	0.02	0.02	0.04	0.06	0.04	0.07
	(1)	(2)	(2)	(3)	(0)	(0)	(1)	(2)	(0)	(0)	(0)	(0)	(0)	(0)	(0)	(0)	(1)	(1)
**Total contribution of I-8**	0.25	0.18	3.66	4.44	0.06	0.03	4.65	4.30	0.81	0.22	1.67	1.60	1.18	1.14	11.08	11.61	2.64	2.40
	(5)	(5)	(34)	(36)	(2)	(1)	(31)	(31)	(8)	(4)	(22)	(26)	(16)	(15)	(52)	(53)	(33)	(36)
Childhood-cluster diseases	0.01	0.01	0.11	0.15	0	0	0.27	0.29	0	0	0.01	0.01	0.05	0.05	0.42	0.48	0.07	0.07
	(0)	(0)	(1)	(1)	(0)	(0)	(2)	(2)	(0)	(0)	(0)	(0)	(1)	(1)	(2)	(2)	(1)	(1)
Diarrheal diseases	0.01	0	0.92	1.44	0.01	0.01	0.59	0.57	-0.01	-0.02	0.09	0.10	0.06	0.05	1.48	1.44	0.28	0.35
	(0)	(0)	(9)	(12)	(0)	(0)	(4)	(4)	(0)	(0)	(1)	(2)	(1)	(1)	(7)	(7)	(3)	(5)
HIV/AIDS	0.06	0.04	0.11	0.06	0.04	0.02	0.18	0.05	0.36	0.15	0.23	0.11	0.02	0.01	1.58	1.75	0.19	0.15
	(1)	(1)	(1)	(0)	(1)	(0)	(1)	(0)	(4)	(3)	(3)	(2)	(0)	(0)	(7)	(8)	(2)	(2)
Lower respiratory infections	0.04	0.01	0.66	0.90	-0.06	-0.07	0.77	0.60	0.28	0.01	0.76	0.84	0.38	0.36	2.21	2.13	0.73	0.62
	(1)	(0)	(6)	(7)	(-2)	(-2)	(5)	(4)	(3)	(0)	(10)	(14)	(5)	(5)	(10)	(10)	(9)	(9)
Malaria	0	0	0.02	0.02	0	0	0.01	0.01	0	0	0	0	0.01	0.01	1.32	1.31	0.01	0.02
	(0)	(0)	(0)	(0)	(0)	(0)	(0)	(0)	(0)	(0)	(0)	(0)	(0)	(0)	(6)	(6)	(0)	(0)
Maternal conditions	0	0.01	0	0.11	0	0.01	0	0.37	0	0	0	0.08	0	0.06	0	1.23	0	0.13
	(0)	(0)	(0)	(1)	(0)	(0)	(0)	(3)	(0)	(0)	(0)	(1)	(0)	(1)	(0)	(6)	(0)	(2)
Neonatal conditions	0.08	0.08	1.12	1.21	0.08	0.07	2.19	1.97	0.02	0.02	0.46	0.39	0.59	0.52	1.71	1.47	0.69	0.60
	(2)	(2)	(10)	(10)	(2)	(2)	(14)	(14)	(0)	(0)	(6)	(6)	(8)	(7)	(8)	(7)	(9)	(9)
Tuberculosis	0.06	0.03	0.73	0.55	0	0	0.64	0.45	0.16	0.04	0.12	0.06	0.07	0.07	2.37	1.80	0.67	0.46
	(1)	(1)	(7)	(4)	(0)	(0)	(4)	(3)	(2)	(1)	(2)	(1)	(1)	(1)	(11)	(8)	(8)	(7)
**Total contribution of other causes**	-1.16	-1.54	0.70	1.63	1.75	1.45	2.83	2.23	2.36	0.08	3.27	1.66	1.23	1.14	5.43	5.08	0.82	0.26
	(-25)	(-41)	(7)	(13)	(51)	(48)	(19)	(16)	(24)	(2)	(43)	(27)	(16)	(15)	(26)	(23)	(10)	(4)
Cardiovascular diseases ex. NCD-7	-0.18	-0.19	-0.09	0.14	0.22	0.11	0.32	0.37	0.76	0.25	0.18	0.17	0.29	0.54	0.38	0.91	-0.05	-0.05
	(-4)	(-5)	(-1)	(1)	(6)	(4)	(2)	(3)	(8)	(5)	(2)	(3)	(4)	(7)	(2)	(4)	(-1)	(-1)
Congenital anomalies	0.05	0.06	0.16	0.19	0.05	0.04	0.37	0.36	0.05	0.04	0.22	0.20	0.26	0.25	0.22	0.22	0.24	0.20
	(1)	(2)	(2)	(2)	(1)	(1)	(2)	(3)	(1)	(1)	(3)	(3)	(4)	(3)	(1)	(1)	(3)	(3)
Digestive diseases ex. NCD-7	-0.20	-0.20	0.46	0.31	0.02	0.06	0.10	0.36	0.57	0.27	0.57	0.39	-0.14	-0.03	0.67	0.48	0.20	0.13
	(-4)	(-5)	(4)	(2)	(1)	(2)	(1)	(3)	(6)	(5)	(7)	(6)	(-2)	(0)	(3)	(2)	(2)	(2)
Endocrine, blood, immune disorders	-0.10	-0.12	-0.10	-0.10	0.12	0.11	-0.02	-0.02	-0.06	-0.08	0.06	0.08	-0.04	-0.04	0.07	0.21	-0.03	-0.03
	(-2)	(-3)	(-1)	(-1)	(4)	(4)	(0)	(0)	(-1)	(-1)	(1)	(1)	(-1)	(0)	(0)	(1)	(0)	(0)
Genitourinary diseases ex. NCD-7	-0.04	-0.09	0.21	0.20	0.12	0.12	0.32	0.38	0.07	0.02	0.39	0.42	0.37	0.46	0.60	0.50	0.23	0.22
	(-1)	(-2)	(2)	(2)	(3)	(4)	(2)	(3)	(1)	(0)	(5)	(7)	(5)	(6)	(3)	(2)	(3)	(3)
Infectious & parasitic diseases ex. I-8	-0.07	-0.05	0.24	0.30	0.10	0.10	0.36	0.37	-0.03	-0.06	0.20	0.22	0.01	-0.01	1.02	0.94	0.16	0.14

© 2025 Karlsson O et al. *JAMA Network Open*.

	China		India		United States		Central Asia		Central & Eastern Europe		Latin America & Caribbean		Middle East & North Africa		Saharan Africa		Western Pacific & Southeast Asia	
	M	F	M	F	M	F	M	F	M	F	M	F	M	F	M	F	M	F
	(-1)	(-1)	(2)	(2)	(3)	(3)	(2)	(3)	(0)	(-1)	(3)	(4)	(0)	(0)	(5)	(4)	(2)	(2)
Violence	-0.01	0.01	0.12	0.04	0.30	0.06	0.73	0.15	0.15	0.05	1.51	0.20	0.64	0.19	0.83	0.24	0.18	0.05
	(0)	(0)	(1)	(0)	(9)	(2)	(5)	(1)	(1)	(1)	(20)	(3)	(9)	(3)	(4)	(1)	(2)	(1)
Cancers ex. NCD-7	-0.32	-0.68	-0.96	-0.61	-0.28	-0.19	-0.18	-0.26	0.37	0.09	-0.15	-0.04	0.05	0.06	0.42	0.51	-0.30	-0.30
	(-7)	(-18)	(-9)	(-5)	(-8)	(-6)	(-1)	(-2)	(4)	(2)	(-2)	(-1)	(1)	(1)	(2)	(2)	(-4)	(-5)
Mental & substance use disorders	-0.16	-0.07	-0.11	-0.06	0.68	0.34	-0.11	-0.05	0.25	0.04	-0.08	-0.06	-0.17	-0.07	-0.10	-0.04	-0.15	-0.07
	(-3)	(-2)	(-1)	(-1)	(20)	(11)	(-1)	(0)	(3)	(1)	(-1)	(-1)	(-2)	(-1)	(0)	(0)	(-2)	(-1)
Musculoskeletal diseases	-0.03	-0.03	-0.01	0.01	0	0	-0.01	0.01	-0.02	-0.03	0.01	0.04	-0.03	-0.04	-0.02	-0.01	-0.01	-0.01
	(-1)	(-1)	(0)	(0)	(0)	(0)	(0)	(0)	(0)	(-1)	(0)	(1)	(0)	(0)	(0)	(0)	(0)	(0)
Neurological conditions	-0.25	-0.13	-0.26	-0.26	0.28	0.60	-0.10	-0.07	-0.24	-0.43	-0.25	-0.42	-0.16	-0.23	-0.02	0	-0.29	-0.44
	(-5)	(-3)	(-2)	(-2)	(8)	(20)	(-1)	(0)	(-2)	(-8)	(-3)	(-7)	(-2)	(-3)	(0)	(0)	(-4)	(-7)
Nutritional deficiencies	0	-0.01	0.03	0.06	0.02	0.04	0.04	0.09	-0.01	-0.02	0.10	0.11	0.01	0.01	0.23	0.24	0.06	0.07
	(0)	(0)	(0)	(1)	(1)	(1)	(0)	(1)	(0)	(0)	(1)	(2)	(0)	(0)	(1)	(1)	(1)	(1)
Other neoplasms	-0.08	-0.08	-0.08	-0.07	-0.03	-0.03	-0.06	-0.06	-0.02	-0.04	0	0.02	-0.07	-0.07	-0.06	-0.05	-0.04	-0.04
	(-2)	(-2)	(-1)	(-1)	(-1)	(-1)	(0)	(0)	(0)	(-1)	(0)	(0)	(-1)	(-1)	(0)	(0)	(-1)	(-1)
Respiratory diseases ex. NCD-7	-0.08	-0.09	0.30	0.54	0	0.02	0.19	0.16	-0.07	-0.09	0.09	0.13	0.01	0.04	0.14	0.22	0.13	0.15
	(-2)	(-2)	(3)	(4)	(0)	(1)	(1)	(1)	(-1)	(-2)	(1)	(2)	(0)	(1)	(1)	(1)	(2)	(2)
Respiratory infections ex. I-8	0	0	0	0	0	0	0	0.01	0	0	0	0	0	0	0.03	0.04	0	0
	(0)	(0)	(0)	(0)	(0)	(0)	(0)	(0)	(0)	(0)	(0)	(0)	(0)	(0)	(0)	(0)	(0)	(0)
Skin diseases	-0.02	-0.03	-0.01	-0.01	0	0	0.01	-0.01	0.01	0	0.05	0.07	0	0	0.03	0.03	0.03	0.03
	(0)	(-1)	(0)	(0)	(0)	(0)	(0)	(0)	(0)	(0)	(1)	(1)	(0)	(0)	(0)	(0)	(0)	(0)
Sudden infant death syndrome	-0.01	0	0.01	0.01	0.02	0.02	0.01	0.01	0	0	0	0	0.01	0.01	0.01	0.01	0.02	0
	(0)	(0)	(0)	(0)	(1)	(1)	(0)	(0)	(0)	(0)	(0)	(0)	(0)	(0)	(0)	(0)	(0)	(0)
Unintentional injuries ex. NCD-7	0.33	0.16	0.79	0.94	0.12	0.06	0.85	0.44	0.58	0.06	0.35	0.12	0.19	0.05	0.98	0.63	0.47	0.22
	(7)	(4)	(7)	(8)	(4)	(2)	(6)	(3)	(6)	(1)	(5)	(2)	(3)	(1)	(5)	(3)	(6)	(3)

Note: Number of years are shown (percentage of the total gap in parentheses below). Comparisons were made to the North Atlantic in 2019 (which had a life expectancy of 80 for males and 84 for females). Negative contribution suggests that the gap in life expectancy would have been greater had it not been for lower mortality from that cause than in the 2019 North Atlantic. Therefore, the contribution of some causes or groups of causes can be greater than the total life expectancy gap. I-8 = 8 priority infectious and maternal and child health conditions. NCD-7 = 7 priority noncommunicable diseases (NCD) and injuries. ex. = excluding. CVD = cardiovascular diseases.

© 2025 Karlsson O et al. *JAMA Network Open*.

eTable 7. Contributions of Causes of Death to Life Expectancy Gaps Relative to the North Atlantic, 2019: Removing Negative Contributions

	China	India	United States	Central Asia	Central & Eastern Europe	Latin America & Caribbean	Middle East & North Africa	Sub-Saharan Africa	Western Pacific & Southeast Asia
**Total life expectancy gap**	4.32	11.54	3.31	14.73	7.61	7.01	7.67	21.61	7.45
**Total contribution of NCD-7**	3.94 (91)	5.69 (49)	1.48 (45)	7.41 (50)	5.46 (72)	2.72 (39)	5.00 (65)	4.99 (23)	3.89 (52)
	[3.70, 3.97]	[5.30, 5.92]	[1.15, 1.76]	[6.89, 7.74]	[5.32, 5.56]	[2.65, 2.76]	[4.75, 5.15]	[4.78, 5.02]	[3.74, 3.95]
Atherosclerotic CVDs	1.33 (31)	2.21 (19)	0.65 (20)	4.16 (28)	4.24 (56)	1.10 (16)	3.28 (43)	1.47 (7)	1.25 (17)
	[1.17, 1.44]	[2.03, 2.32]	[0.44, 0.84]	[3.82, 4.38]	[4.11, 4.32]	[1.05, 1.14]	[3.09, 3.38]	[1.37, 1.51]	[1.15, 1.30]
Diabetes	0.10 (2)	0.53 (5)	0.17 (5)	0.68 (5)	0.11 (1)	0.77 (11)	0.51 (7)	0.79 (4)	0.42 (6)
	[0.08, 0.11]	[0.48, 0.56]	[0.15, 0.20]	[0.62, 0.74]	[0.10, 0.11]	[0.75, 0.79]	[0.48, 0.54]	[0.76, 0.81]	[0.40, 0.44]
Infection-related NCDs	0.47 (11)	0.50 (4)	0 (0)	0.85 (6)	0.29 (4)	0.23 (3)	0.54 (7)	0.80 (4)	0.57 (8)
	[0.43, 0.51]	[0.45, 0.55]	[na]	[0.79, 0.93]	[0.28, 0.31]	[0.22, 0.24]	[0.51, 0.58]	[0.78, 0.82]	[0.55, 0.60]
Tobacco-related NCDs	0.85 (20)	1.40 (12)	0.25 (7)	0.52 (3)	0.07 (1)	0 (0)	0.05 (1)	0 (0)	0.25 (3)
	[0.74, 0.94]	[1.24, 1.54]	[0.17, 0.32]	[0.39, 0.65]	[0.06, 0.10]	[na]	[0.01, 0.09]	[na]	[0.22, 0.29]
Hemorrhagic stroke	0.93 (22)	0.61 (5)	0.05 (1)	0.90 (6)	0.36 (5)	0.30 (4)	0.28 (4)	1.05 (5)	1.05 (14)
	[0.84, 1.00]	[0.55, 0.67]	[0.03, 0.06]	[0.81, 1.00]	[0.35, 0.37]	[0.29, 0.31]	[0.26, 0.31]	[1.00, 1.08]	[0.99, 1.09]
Road injury	0.25 (6)	0.29 (3)	0.21 (6)	0.30 (2)	0.18 (2)	0.32 (5)	0.34 (4)	0.75 (3)	0.34 (5)
	[0.22, 0.26]	[0.27, 0.30]	[0.20, 0.22]	[0.27, 0.31]	[0.18, 0.19]	[0.31, 0.32]	[0.32, 0.34]	[0.70, 0.74]	[0.32, 0.35]
Suicide	0 (0)	0.15 (1)	0.15 (4)	0 (0)	0.20 (3)	0 (0)	0 (0)	0.12 (1)	0 (0)
	[na]	[0.13, 0.17]	[0.14, 0.16]	[0, 0.01]	[0.19, 0.20]	[na]	[na]	[0.11, 0.13]	[na]
**Total contribution of I-8**	0.16 (4)	3.63 (31)	0.11 (3)	4.39 (30)	0.49 (7)	1.52 (22)	1.06 (14)	11.27 (52)	2.25 (30)
	[0.12, 0.20]	[3.33, 3.96]	[0.09, 0.12]	[4.03, 4.80]	[0.48, 0.51]	[1.47, 1.55]	[0.98, 1.15]	[10.79, 11.62]	[2.14, 2.33]
Childhood-cluster diseases	0.01 (0)	0.12 (1)	0 (0)	0.27 (2)	0 (0)	0.01 (0)	0.04 (1)	0.44 (2)	0.06 (1)
	[0, 0.01]	[0.07, 0.23]	[na]	[0.20, 0.47]	[0, 0]	[0.01, 0.02]	[0.03, 0.08]	[0.39, 0.60]	[0.06, 0.09]
Diarrheal diseases	0 (0)	1.05 (9)	0.01 (0)	0.57 (4)	0 (0)	0.09 (1)	0.05 (1)	1.45 (7)	0.28 (4)
	[0, 0.01]	[0.88, 1.30]	[0.01, 0.01]	[0.47, 0.71]	[na]	[0.08, 0.09]	[0.03, 0.08]	[1.38, 1.58]	[0.25, 0.31]
HIV/AIDS	0.04 (1)	0.08 (1)	0.02 (1)	0.12 (1)	0.25 (3)	0.16 (2)	0.02 (0)	1.65 (8)	0.15 (2)
	[0.03, 0.04]	[0.06, 0.09]	[0.02, 0.02]	[0.09, 0.22]	[0.24, 0.25]	[0.15, 0.16]	[0.02, 0.02]	[1.58, 1.69]	[0.14, 0.16]
Lower respiratory infections	0.02 (0)	0.70 (6)	0 (0)	0.66 (5)	0.13 (2)	0.74 (11)	0.34 (4)	2.16 (10)	0.61 (8)
	[0, 0.05]	[0.60, 0.78]	[na]	[0.56, 0.78]	[0.12, 0.14]	[0.71, 0.77]	[0.30, 0.40]	[2.01, 2.27]	[0.56, 0.65]
Malaria	0 (0)	0.02 (0)	0 (0)	0.01 (0)	0 (0)	0 (0)	0.01 (0)	1.30 (6)	0.01 (0)
	[na]	[0.02, 0.02]	[na]	[0, 0.01]	[na]	[0, 0]	[0.01, 0.02]	[1.15, 1.47]	[0.01, 0.02]
Maternal conditions	0 (0)	0.05 (0)	0.01 (0)	0.21 (1)	0 (0)	0.04 (1)	0.02 (0)	0.60 (3)	0.05 (1)
	[0, 0]	[0.04, 0.05]	[0, 0.01]	[0.18, 0.24]	[0, 0]	[0.03, 0.04]	[0.02, 0.03]	[0.56, 0.64]	[0.05, 0.06]
Neonatal conditions	0.06 (1)	1.04 (9)	0.07 (2)	2.02 (14)	0.02 (0)	0.39 (6)	0.51 (7)	1.58 (7)	0.57 (8)
	[0.03, 0.08]	[0.82, 1.19]	[0.05, 0.09]	[1.67, 2.29]	[0, 0.03]	[0.36, 0.42]	[0.45, 0.56]	[1.39, 1.72]	[0.49, 0.64]

© 2025 Karlsson O et al. *JAMA Network Open*.

	China	India	United States	Central Asia	Central & Eastern Europe	Latin America & Caribbean	Middle East & North Africa	Sub-Saharan Africa	Western Pacific & Southeast Asia
Tuberculosis	0.03 (1) [0.03, 0.03]	0.59 (5) [0.54, 0.62]	0 (0) [na]	0.53 (4) [0.49, 0.55]	0.10 (1) [0.09, 0.10]	0.08 (1) [0.08, 0.09]	0.06 (1) [0.06, 0.07]	2.08 (10) [1.97, 2.12]	0.51 (7) [0.49, 0.52]
**Total contribution of other causes**	0.23 (5) [0.18, 0.47]	2.22 (19) [1.86, 2.75]	1.73 (52) [1.44, 2.07]	2.93 (20) [2.41, 3.57]	1.66 (22) [1.55, 1.81]	2.78 (40) [2.72, 2.87]	1.61 (21) [1.43, 1.87]	5.36 (25) [5.07, 5.94]	1.31 (18) [1.24, 1.51]

Note: Number of years are shown (percentage of the total gap in parentheses) with 95% uncertainty bounds in square brackets below. Comparisons were made to the 2019 North Atlantic (which had a life expectancy of 82 years). To facilitate the presentation of results, causes that had a negative contribution—which occurred when the target location had achieved lower cause-specific mortality rates than the North Atlantic—were removed. However, after this adjustment, adding the contribution of all causes together could result in a total contribution greater than the actual life expectancy gap. Therefore, we projected the estimated proportional contribution of each cause (after removing negative impacts) back onto the life expectancy gap. I-8 = 8 priority infectious and maternal and child health conditions. NCD-7 = 7 priority noncommunicable diseases (NCD) and injuries. CVD = cardiovascular diseases.

© 2025 Karlsson O et al. *JAMA Network Open*.

eAppendix 3. Arriaga's Decomposition Method

Arriaga's method[6,7] is done in two steps. First, the contribution of all-cause mortality at each age to the life expectancy gap is calculated. Using a life table with a radix of one the contribution w_x at age *x* is calculated as:

$$w_x = \underbrace{l_x\left[\frac{{}_n\ddot{L}_x}{\ddot{l}_x} - \frac{{}_nL_x}{l_x}\right]}_{direct\ effect} + \underbrace{\left[l_x\frac{\ddot{l}_{x+n}}{\ddot{l}_x} - l_{x+n}\right]\ddot{e}_{x+n}}_{indirect\ effect}$$

where ${}_nL_x$ is the person-years of life contributed at age x to $x+n$ and l_x is the proportion surviving to age x in the target location, with two dots over a letter indicating the same estimates for the North Atlantic. $\ddot{e}_x$ is the life expectancy at age x in the North Atlantic. The first term shows the direct effect, and the rest shows the indirect effect. The direct effect is the gap that arises strictly within the corresponding age interval, and the indirect effect is the additional gap due to changes in the number of survivors at the end of the age interval. The indirect effect is excluded for the last (open ended) age interval 85+.

The contribution of each cause of death is then obtained in the second step:

$$C_i = \sum_{x=0}^{85+} w_x \frac{{}_n\ddot{m}_{x,i} - {}_nm_{x,i}}{{}_n\ddot{M}_x - {}_nM_x}$$

where ${}_nm_{x,i}$ is the cause-specific mortality rate for cause i at age x to $x+n$ and ${}_nM_x$ is the all-cause mortality rate. C_i is the contribution of cause i to the life expectancy gap between the North Atlantic and the target location. As for Pollard's method, summing up C_i over all causes of deaths shows the total gap in life expectancy.

© 2025 Karlsson O et al. *JAMA Network Open*.

eTable 8. Contributions of Causes of Death to Life Expectancy Gaps Relative to the North Atlantic, 2019: Arriaga's Method

	China	India	United States	Central Asia	Central & Eastern Europe	Latin America & Caribbean	Middle East & North Africa	Sub-Saharan Africa	Western Pacific & Southeast Asia
**Total life expectancy gap**	4.29	11.48	3.31	14.62	7.58	6.98	7.61	21.51	7.43
**Total contribution of NCD-7**	5.33 (124)	6.06 (53)	1.60 (48)	7.06 (48)	5.53 (73)	2.75 (39)	5.10 (67)	4.49 (21)	4.22 (57)
	[4.83, 5.85]	[5.70, 6.41]	[1.29, 1.86]	[6.59, 7.43]	[5.40, 5.64]	[2.67, 2.83]	[4.88, 5.27]	[4.30, 4.53]	[4.04, 4.38]
Atherosclerotic CVDs	1.80 (42)	2.40 (21)	0.70 (21)	3.83 (26)	4.14 (55)	1.14 (16)	3.40 (45)	1.25 (6)	1.34 (18)
	[1.55, 2.07]	[2.23, 2.55]	[0.50, 0.88]	[3.52, 4.07]	[4.03, 4.22]	[1.09, 1.18]	[3.22, 3.50]	[1.17, 1.28]	[1.24, 1.42]
Diabetes	0.14 (3)	0.56 (5)	0.19 (6)	0.68 (5)	0.12 (2)	0.81 (12)	0.54 (7)	0.69 (3)	0.46 (6)
	[0.11, 0.17]	[0.52, 0.60]	[0.17, 0.21]	[0.62, 0.74]	[0.11, 0.12]	[0.79, 0.83]	[0.51, 0.57]	[0.66, 0.70]	[0.44, 0.48]
Infection-related NCDs	0.67 (16)	0.56 (5)	-0.01 (0)	0.88 (6)	0.33 (4)	0.25 (4)	0.60 (8)	0.78 (4)	0.63 (8)
	[0.61, 0.74]	[0.51, 0.62]	[-0.02, 0]	[0.83, 0.96]	[0.32, 0.35]	[0.24, 0.26]	[0.56, 0.64]	[0.76, 0.80]	[0.61, 0.66]
Tobacco-related NCDs	1.13 (26)	1.35 (12)	0.26 (8)	0.46 (3)	0.12 (2)	-0.09 (-1)	0.04 (1)	-0.07 (0)	0.26 (4)
	[0.95, 1.32]	[1.20, 1.50]	[0.19, 0.33]	[0.36, 0.59]	[0.10, 0.14]	[-0.10, -0.06]	[0, 0.09]	[-0.09, -0.05]	[0.22, 0.30]
Hemorrhagic stroke	1.29 (30)	0.69 (6)	0.05 (2)	0.90 (6)	0.39 (5)	0.32 (5)	0.30 (4)	0.95 (4)	1.15 (15)
	[1.14, 1.43]	[0.62, 0.76]	[0.04, 0.07]	[0.81, 1.00]	[0.38, 0.40]	[0.31, 0.33]	[0.28, 0.34]	[0.91, 0.98]	[1.08, 1.20]
Road injury	0.36 (8)	0.34 (3)	0.24 (7)	0.32 (2)	0.21 (3)	0.36 (5)	0.38 (5)	0.77 (4)	0.40 (5)
	[0.34, 0.37]	[0.32, 0.35]	[0.23, 0.25]	[0.29, 0.33]	[0.20, 0.21]	[0.35, 0.36]	[0.36, 0.38]	[0.72, 0.76]	[0.37, 0.39]
Suicide	-0.06 (-1)	0.18 (2)	0.17 (5)	-0.01 (0)	0.22 (3)	-0.04 (-1)	-0.16 (-2)	0.12 (1)	-0.01 (0)
	[-0.07, -0.04]	[0.16, 0.20]	[0.16, 0.17]	[-0.04, 0.01]	[0.22, 0.23]	[-0.04, -0.03]	[-0.17, -0.16]	[0.11, 0.13]	[-0.02, 0]
**Total contribution of I-8**	0.23 (5)	3.98 (35)	0.06 (2)	4.72 (32)	0.58 (8)	1.62 (23)	1.20 (16)	11.63 (54)	2.51 (34)
	[0.18, 0.29]	[3.70, 4.38]	[0.03, 0.09]	[4.34, 5.18]	[0.56, 0.60]	[1.57, 1.66]	[1.11, 1.31]	[11.11, 12.03]	[2.42, 2.62]
Childhood-cluster diseases	0.01 (0)	0.14 (1)	0 (0)	0.30 (2)	0 (0)	0.01 (0)	0.05 (1)	0.49 (2)	0.07 (1)
	[0.01, 0.02]	[0.08, 0.27]	[0, 0]	[0.21, 0.52]	[0, 0]	[0.01, 0.02]	[0.03, 0.09]	[0.43, 0.66]	[0.07, 0.10]
Diarrheal diseases	0.01 (0)	1.04 (9)	0.01 (0)	0.56 (4)	-0.01 (0)	0.09 (1)	0.06 (1)	1.44 (7)	0.31 (4)
	[0, 0.01]	[0.89, 1.28]	[0.01, 0.01]	[0.48, 0.69]	[-0.01, -0.01]	[0.09, 0.10]	[0.04, 0.09]	[1.35, 1.57]	[0.28, 0.35]
HIV/AIDS	0.05 (1)	0.09 (1)	0.03 (1)	0.13 (1)	0.28 (4)	0.18 (3)	0.02 (0)	1.76 (8)	0.17 (2)
	[0.05, 0.06]	[0.08, 0.11]	[0.03, 0.03]	[0.10, 0.24]	[0.28, 0.29]	[0.17, 0.18]	[0.02, 0.02]	[1.68, 1.80]	[0.17, 0.18]
Lower respiratory infections	0.03 (1)	0.77 (7)	-0.06 (-2)	0.73 (5)	0.17 (2)	0.76 (11)	0.38 (5)	2.12 (10)	0.65 (9)
	[0, 0.07]	[0.67, 0.88]	[-0.08, -0.04]	[0.61, 0.86]	[0.16, 0.18]	[0.73, 0.79]	[0.33, 0.45]	[1.97, 2.25]	[0.60, 0.70]
Malaria	0 (0)	0.02 (0)	0 (0)	0.01 (0)	0 (0)	0 (0)	0.01 (0)	1.42 (7)	0.01 (0)
	[na]	[0.02, 0.03]	[na]	[0.01, 0.01]	[na]	[0, 0]	[0.01, 0.02]	[1.24, 1.61]	[0.01, 0.02]
Maternal conditions	0 (0)	0.05 (0)	0.01 (0)	0.23 (2)	0 (0)	0.04 (1)	0.03 (0)	0.65 (3)	0.06 (1)
	[0, 0]	[0.05, 0.06]	[0, 0.01]	[0.19, 0.27]	[0, 0]	[0.04, 0.04]	[0.02, 0.03]	[0.61, 0.69]	[0.05, 0.07]
Neonatal conditions	0.08 (2)	1.22 (11)	0.08 (2)	2.22 (15)	0.02 (0)	0.45 (6)	0.58 (8)	1.77 (8)	0.67 (9)
	[0.04, 0.12]	[0.98, 1.41]	[0.06, 0.10]	[1.84, 2.53]	[0, 0.03]	[0.41, 0.48]	[0.50, 0.64]	[1.56, 1.93]	[0.58, 0.75]
Tuberculosis	0.05 (1)	0.65 (6)	0 (0)	0.55 (4)	0.11 (1)	0.09 (1)	0.07 (1)	1.98 (9)	0.56 (8)

© 2025 Karlsson O et al. *JAMA Network Open*.

	China	India	United States	Central Asia	Central & Eastern Europe	Latin America & Caribbean	Middle East & North Africa	Sub-Saharan Africa	Western Pacific & Southeast Asia
	[0.04, 0.05]	[0.60, 0.70]	[0, 0]	[0.52, 0.58]	[0.11, 0.11]	[0.09, 0.09]	[0.07, 0.07]	[1.88, 2.02]	[0.54, 0.57]
**Total contribution of other causes**	-1.27 (-30)<br>[-1.80, -0.77]	1.44 (13)<br>[0.88, 1.92]	1.66 (50)<br>[1.37, 1.98]	2.84 (19)<br>[2.26, 3.45]	1.47 (19)<br>[1.35, 1.61]	2.61 (37)<br>[2.52, 2.72]	1.31 (17)<br>[1.10, 1.57]	5.39 (25)<br>[5.05, 6.01]	0.69 (9)<br>[0.48, 0.93]

Note: Number of years are shown (percentage of the total gap in parentheses) with 95% uncertainty bounds in square brackets below. Comparisons were made to the 2019 North Atlantic (which had a life expectancy of 82 years). Negative contribution suggests that the gap in life expectancy would have been greater had it not been for lower mortality from that cause than in the 2019 North Atlantic. Therefore, the contribution of some causes or groups of causes can be over 100% of the total life expectancy gap. I-8 = 8 priority infectious and maternal and child health conditions. NCD-7 = 7 priority noncommunicable diseases (NCD) and injuries. CVD = cardiovascular diseases.

© 2025 Karlsson O et al. *JAMA Network Open*.

eTable 9. Contributions of Causes of Death to Life Expectancy Gaps Relative to the Top Life Expectancy Decile, 2019

	Decile 9	Decile 8	Decile 7	Decile 6	Decile 5	Decile 4	Decile 3	Decile 2	Decile 1
**Total life expectancy gap**	2.99	6.77	8.59	10.48	11.85	13.99	17.44	20.20	27.83
**Total contribution of NCD-7**	1.43 (48) [1.27, 1.56]	3.88 (57) [3.69, 4.02]	4.36 (51) [4.20, 4.50]	7.27 (69) [7.00, 7.48]	7.64 (64) [7.18, 7.99]	7.53 (54) [7.10, 7.92]	7.04 (40) [6.64, 7.33]	5.40 (27) [5.15, 5.50]	5.77 (21) [5.43, 5.94]
Atherosclerotic CVDs	0.96 (32) [0.85, 1.05]	2.10 (31) [1.96, 2.18]	2.71 (32) [2.59, 2.80]	4.84 (46) [4.62, 4.98]	4.42 (37) [4.11, 4.63]	3.40 (24) [3.15, 3.61]	3.02 (17) [2.77, 3.20]	1.73 (9) [1.61, 1.79]	1.98 (7) [1.79, 2.09]
Diabetes	0.17 (6) [0.16, 0.19]	0.53 (8) [0.50, 0.55]	0.81 (9) [0.79, 0.83]	0.36 (3) [0.34, 0.38]	0.79 (7) [0.73, 0.85]	0.78 (6) [0.73, 0.83]	1.00 (6) [0.93, 1.05]	0.88 (4) [0.83, 0.90]	0.79 (3) [0.74, 0.83]
Infection-related NCDs	-0.09 (-3) [-0.10, -0.08]	0.21 (3) [0.19, 0.24]	0.11 (1) [0.10, 0.12]	0.35 (3) [0.33, 0.39]	0.88 (7) [0.82, 0.97]	0.64 (5) [0.61, 0.70]	0.74 (4) [0.70, 0.81]	0.75 (4) [0.73, 0.78]	0.82 (3) [0.78, 0.87]
Tobacco-related NCDs	0.33 (11) [0.30, 0.37]	0.39 (6) [0.35, 0.44]	0.21 (2) [0.18, 0.24]	0.33 (3) [0.29, 0.38]	0.41 (3) [0.32, 0.52]	0.62 (4) [0.55, 0.70]	0.79 (5) [0.68, 0.89]	0.10 (0) [0.07, 0.14]	0.11 (0) [0.06, 0.16]
Hemorrhagic stroke	0.01 (0) [0, 0.02]	0.36 (5) [0.34, 0.39]	0.29 (3) [0.28, 0.31]	0.83 (8) [0.78, 0.88]	0.91 (8) [0.82, 1.02]	1.90 (14) [1.74, 2.07]	1.02 (6) [0.94, 1.11]	1.22 (6) [1.15, 1.26]	1.09 (4) [1.00, 1.15]
Road injury	0.10 (3) [0.09, 0.10]	0.38 (6) [0.37, 0.38]	0.35 (4) [0.34, 0.36]	0.44 (4) [0.43, 0.45]	0.39 (3) [0.37, 0.39]	0.36 (3) [0.34, 0.37]	0.50 (3) [0.47, 0.51]	0.70 (3) [0.66, 0.69]	0.88 (3) [0.81, 0.88]
Suicide	-0.06 (-2) [-0.06, -0.05]	-0.09 (-1) [-0.10, -0.08]	-0.12 (-1) [-0.13, -0.11]	0.13 (1) [0.12, 0.14]	-0.17 (-1) [-0.18, -0.16]	-0.16 (-1) [-0.17, -0.16]	-0.04 (0) [-0.05, -0.02]	0.02 (0) [0.01, 0.03]	0.11 (0) [0.09, 0.13]
**Total contribution of I-8**	0.11 (4) [0.07, 0.15]	1.08 (16) [1.02, 1.13]	1.25 (15) [1.18, 1.31]	1.06 (10) [1.00, 1.13]	2.05 (17) [1.91, 2.25]	3.60 (26) [3.45, 3.78]	6.38 (37) [6.07, 6.70]	9.41 (47) [9.08, 9.60]	14.51 (52) [13.59, 15.40]
Childhood-cluster diseases	0 (0) [0, 0]	0.01 (0) [0.01, 0.02]	0.01 (0) [0, 0.01]	0.02 (0) [0.01, 0.02]	0.03 (0) [0.03, 0.05]	0.10 (1) [0.09, 0.14]	0.24 (1) [0.19, 0.37]	0.25 (1) [0.22, 0.35]	0.79 (3) [0.63, 1.17]
Diarrheal diseases	0.01 (0) [0.01, 0.01]	0.04 (1) [0.04, 0.06]	0.05 (1) [0.05, 0.06]	0.04 (0) [0.03, 0.05]	0.35 (3) [0.28, 0.51]	0.55 (4) [0.49, 0.66]	0.78 (4) [0.71, 0.91]	1.15 (6) [1.09, 1.24]	2.02 (7) [1.84, 2.30]
HIV/AIDS	0.01 (0) [0.01, 0.01]	0.15 (2) [0.14, 0.15]	0.11 (1) [0.11, 0.11]	0.31 (3) [0.30, 0.33]	0.04 (0) [0.03, 0.04]	0.29 (2) [0.27, 0.30]	0.74 (4) [0.70, 0.83]	1.68 (8) [1.59, 1.73]	1.39 (5) [1.28, 1.48]
Lower respiratory infections	0.01 (0) [-0.03, 0.04]	0.50 (7) [0.45, 0.54]	0.50 (6) [0.46, 0.53]	0.10 (1) [0.06, 0.14]	0.44 (4) [0.37, 0.52]	0.82 (6) [0.76, 0.89]	0.99 (6) [0.88, 1.09]	1.79 (9) [1.67, 1.88]	2.76 (10) [2.49, 3.03]
Malaria	0 (0) [na]	0 (0) [0, 0]	0 (0) [0, 0]	0 (0) [0, 0]	0 (0) [0, 0]	0.04 (0) [0.03, 0.04]	0.30 (2) [0.27, 0.33]	0.76 (4) [0.71, 0.79]	1.90 (7) [1.56, 2.33]
Maternal conditions	0 (0) [0, 0]	0.01 (0) [0.01, 0.01]	0.03 (0) [0.03, 0.03]	0.02 (0) [0.02, 0.03]	0.05 (0) [0.04, 0.05]	0.09 (1) [0.08, 0.11]	0.21 (1) [0.19, 0.24]	0.41 (2) [0.38, 0.45]	1.18 (4) [1.07, 1.30]
Neonatal conditions	0.08 (3) [0.06, 0.09]	0.28 (4) [0.24, 0.30]	0.48 (6) [0.43, 0.52]	0.33 (3) [0.27, 0.38]	0.78 (7) [0.66, 0.89]	0.76 (5) [0.65, 0.85]	1.85 (11) [1.60, 2.05]	1.66 (8) [1.50, 1.79]	1.83 (7) [1.51, 2.14]
Tuberculosis	0 (0)	0.09 (1)	0.08 (1)	0.24 (2)	0.37 (3)	0.96 (7)	1.27 (7)	1.70 (8)	2.63 (9)

© 2025 Karlsson O et al. *JAMA Network Open*.

	Decile 9	Decile 8	Decile 7	Decile 6	Decile 5	Decile 4	Decile 3	Decile 2	Decile 1
	[0, 0]	[0.09, 0.10]	[0.08, 0.08]	[0.23, 0.25]	[0.34, 0.41]	[0.92, 0.98]	[1.19, 1.33]	[1.61, 1.73]	[2.41, 2.75]
**Total contribution of other causes**	1.45 (48)<br>[1.29, 1.64]	1.81 (27)<br>[1.66, 2.03]	2.97 (35)<br>[2.82, 3.18]	2.14 (20)<br>[1.90, 2.43]	2.16 (18)<br>[1.72, 2.67]	2.86 (20)<br>[2.40, 3.37]	4.02 (23)<br>[3.57, 4.57]	5.39 (27)<br>[5.19, 5.86]	7.54 (27)<br>[6.65, 8.64]

Note: Number of years are shown (percentage of the total gap in parentheses) with 95% uncertainty bounds in square brackets below. The global population was divided into deciles according to life expectancy at birth. We excluded the three largest countries—China, India, and the United States—from the deciles, to allow construction of deciles of roughly equal population size (around 500 million) and so that the results for some deciles would not be driven by individual countries. We decomposed the differences between the top decile and other deciles. Comparisons were made to the decile with the greatest life expectancy (84 years). Negative contribution suggests that the gap in life expectancy would have been greater had it not been for lower mortality from that cause than in the top decile. Therefore, the contribution of some causes or groups of causes can be over 100% of the total life expectancy gap. I-8 = 8 priority infectious and maternal and child health conditions. NCD-7 = 7 priority noncommunicable diseases (NCD) and injuries. CVD = cardiovascular diseases.

© 2025 Karlsson O et al. *JAMA Network Open*.

eTable 10. Contributions of Causes of Death to Life Expectancy Gaps Relative to the North Atlantic, 2019: Selected Countries with High or Medium Quality Data

	Brazil	Iran	Jordan	Kazakh-stan	Sri Lanka	Philipp-ines	Russia	Türkiye	South Africa
**Total life expectancy gap**	6.45	5.42	5.41	8.61	5.57	12.62	9.19	4.53	16.20
**Total contribution of NCD-7**	2.43 (38)	4.17 (77)	3.78 (70)	7.07 (82)	5.27 (95)	5.77 (46)	6.71 (73)	4.04 (89)	4.24 (26)
	[2.27, 2.58]	[3.84, 4.47]	[3.24, 4.32]	[6.73, 7.44]	[4.67, 5.99]	[5.54, 6.02]	[6.46, 6.93]	[3.50, 4.62]	[4.00, 4.48]
Atherosclerotic CVDs	1.03 (16)	2.85 (53)	2.19 (41)	4.29 (50)	2.52 (45)	3.08 (24)	5.05 (55)	2.21 (49)	0.88 (5)
	[0.94, 1.13]	[2.61, 3.07]	[1.83, 2.53]	[4.02, 4.53]	[2.12, 2.95]	[2.92, 3.23]	[4.83, 5.24]	[1.84, 2.60]	[0.76, 1.00]
Diabetes	0.55 (9)	0.38 (7)	1.00 (18)	0.04 (0)	1.22 (22)	0.90 (7)	0.18 (2)	0.50 (11)	1.37 (8)
	[0.52, 0.58]	[0.35, 0.43]	[0.87, 1.16]	[0.03, 0.06]	[1.08, 1.39]	[0.85, 0.95]	[0.17, 0.20]	[0.43, 0.59]	[1.29, 1.44]
Infection-related NCDs	0.20 (3)	0.58 (11)	0.19 (3)	0.81 (9)	-0.07 (-1)	0.26 (2)	0.38 (4)	0.27 (6)	0.33 (2)
	[0.19, 0.21]	[0.54, 0.62]	[0.15, 0.24]	[0.75, 0.92]	[-0.09, -0.05]	[0.24, 0.28]	[0.36, 0.41]	[0.23, 0.33]	[0.31, 0.35]
Tobacco-related NCDs	0.05 (1)	-0.17 (-3)	-0.05 (-1)	0.41 (5)	0.50 (9)	0.23 (2)	-0.03 (0)	0.86 (19)	0.15 (1)
	[0.01, 0.09]	[-0.21, -0.12]	[-0.14, 0.08]	[0.35, 0.48]	[0.32, 0.71]	[0.19, 0.29]	[-0.04, 0]	[0.69, 1.07]	[0.10, 0.21]
Hemorrhagic stroke	0.32 (5)	0.03 (1)	0.15 (3)	0.94 (11)	0.78 (14)	1.16 (9)	0.51 (5)	0.31 (7)	0.72 (4)
	[0.31, 0.34]	[0.02, 0.05]	[0.10, 0.21]	[0.88, 1.00]	[0.65, 0.94]	[1.10, 1.21]	[0.49, 0.52]	[0.25, 0.39]	[0.67, 0.77]
Road injury	0.34 (5)	0.59 (11)	0.51 (9)	0.28 (3)	0.20 (4)	0.28 (2)	0.27 (3)	0.05 (1)	0.54 (3)
	[0.33, 0.35]	[0.56, 0.61]	[0.47, 0.54]	[0.27, 0.29]	[0.18, 0.21]	[0.27, 0.29]	[0.26, 0.28]	[0.04, 0.06]	[0.51, 0.56]
Suicide	-0.06 (-1)	-0.10 (-2)	-0.21 (-4)	0.29 (3)	0.12 (2)	-0.13 (-1)	0.35 (4)	-0.16 (-4)	0.26 (2)
	[-0.07, -0.06]	[-0.11, -0.09]	[-0.22, -0.21]	[0.27, 0.31]	[0.08, 0.17]	[-0.13, -0.12]	[0.34, 0.36]	[-0.17, -0.15]	[0.23, 0.30]
**Total contribution of I-8**	1.50 (23)	0.59 (11)	0.60 (11)	0.49 (6)	0.43 (8)	4.00 (32)	0.68 (7)	0.42 (9)	8.07 (50)
	[1.40, 1.58]	[0.48, 0.69]	[0.47, 0.75]	[0.43, 0.55]	[0.34, 0.55]	[3.75, 4.29]	[0.65, 0.71]	[0.31, 0.53]	[7.70, 8.58]
Childhood-cluster diseases	0 (0)	0.01 (0)	0.01 (0)	0 (0)	0.01 (0)	0.15 (1)	0 (0)	0.01 (0)	0.07 (0)
	[0, 0]	[0, 0.02]	[0.01, 0.03]	[0, 0]	[0.01, 0.03]	[0.10, 0.26]	[0, 0]	[0.01, 0.02]	[0.05, 0.15]
Diarrheal diseases	0.05 (1)	0 (0)	0 (0)	-0.01 (0)	0.07 (1)	0.25 (2)	-0.02 (0)	0 (0)	0.79 (5)
	[0.05, 0.06]	[0, 0.01]	[-0.01, 0.01]	[-0.01, -0.01]	[0.05, 0.11]	[0.20, 0.32]	[-0.02, -0.02]	[-0.01, 0]	[0.70, 0.95]
HIV/AIDS	0.14 (2)	0.06 (1)	-0.01 (0)	0.01 (0)	0.01 (0)	0.02 (0)	0.50 (5)	0 (0)	2.23 (14)
	[0.13, 0.14]	[0.06, 0.07]	[-0.01, 0]	[0.01, 0.01]	[0.01, 0.01]	[0.02, 0.02]	[0.49, 0.50]	[0, 0]	[2.14, 2.32]
Lower respiratory infections	0.79 (12)	0.14 (3)	0.19 (4)	0.26 (3)	0.18 (3)	2.08 (17)	0.07 (1)	0.19 (4)	1.53 (9)
	[0.73, 0.85]	[0.10, 0.21]	[0.13, 0.30]	[0.24, 0.29]	[0.12, 0.27]	[1.92, 2.30]	[0.06, 0.09]	[0.13, 0.26]	[1.43, 1.62]
Malaria	0 (0)	0 (0)	0 (0)	0 (0)	0 (0)	0 (0)	0 (0)	0 (0)	0 (0)
	[0, 0]	[na]	[na]	[na]	[na]	[0, 0]	[na]	[na]	[0, 0.01]
Maternal conditions	0.02 (0)	0.01 (0)	0.01 (0)	0.01 (0)	0.01 (0)	0.06 (0)	0 (0)	0 (0)	0.06 (0)
	[0.02, 0.03]	[0.01, 0.01]	[0.01, 0.02]	[0.01, 0.01]	[0.01, 0.01]	[0.05, 0.07]	[0, 0]	[0, 0]	[0.05, 0.07]
Neonatal conditions	0.42 (6)	0.34 (6)	0.39 (7)	0.13 (1)	0.08 (1)	0.68 (5)	-0.01 (0)	0.21 (5)	0.65 (4)
	[0.34, 0.48]	[0.25, 0.42]	[0.27, 0.49]	[0.08, 0.17]	[0.02, 0.13]	[0.54, 0.80]	[-0.03, 0.01]	[0.12, 0.30]	[0.47, 0.81]

© 2025 Karlsson O et al. *JAMA Network Open*.

	Brazil	Iran	Jordan	Kazakh-stan	Sri Lanka	Philipp-ines	Russia	Türkiye	South Africa
Tuberculosis	0.08 (1) [0.07, 0.08]	0.03 (1) [0.03, 0.03]	0 (0) [0, 0]	0.09 (1) [0.08, 0.09]	0.08 (1) [0.07, 0.08]	0.76 (6) [0.74, 0.77]	0.13 (1) [0.12, 0.14]	0.01 (0) [0.01, 0.01]	2.74 (17) [2.48, 3.12]
**Total contribution of other causes**	2.52 (39) [2.32, 2.74]	0.67 (12) [0.32, 1.01]	1.03 (19) [0.41, 1.59]	1.06 (12) [0.65, 1.43]	-0.13 (-2) [-0.89, 0.49]	2.85 (23) [2.44, 3.21]	1.81 (20) [1.57, 2.06]	0.07 (1) [-0.57, 0.61]	3.88 (24) [3.24, 4.33]

Note: Number of years are shown (percentage of the total gap in parentheses) with 95% uncertainty bounds in square brackets below. Comparisons were made to the 2019 North Atlantic (which had a life expectancy of 82 years). Negative contribution suggests that the gap in life expectancy would have been greater had it not been for lower mortality from that cause than in the 2019 North Atlantic. Therefore, the contribution of some causes or groups of causes can be over 100% of the total life expectancy gap. I-8 = 8 priority infectious and maternal and child health conditions. NCD-7 = 7 priority noncommunicable diseases (NCD) and injuries. CVD = cardiovascular diseases.

© 2025 Karlsson O et al. *JAMA Network Open*.

eFigure 1. Contributions of Causes of Death to Life Expectancy Gaps Relative to the North Atlantic: Central Asia and Western Pacific & Southeast Asia, 2019

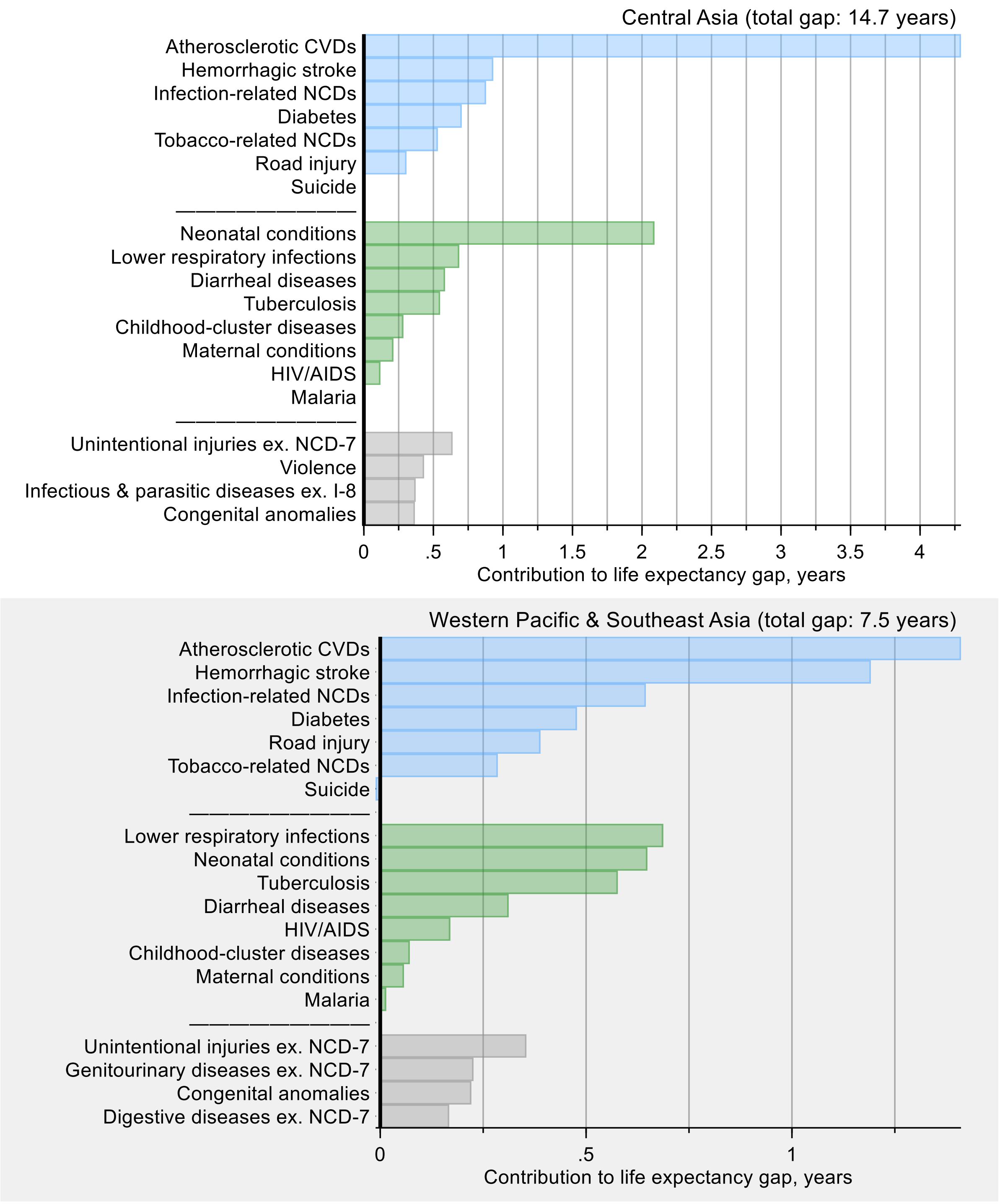

Note: Blue shows NCD-7, green I-8, and gray top 4 other causes (positive impact only). Comparisons were made to the 2019 North Atlantic (which had a life expectancy of 82 years). I-8 = 8 priority infectious and maternal and child health conditions. NCD-7 = 7 priority noncommunicable diseases (NCD) and injuries. ex. = excluding. CVD = cardiovascular diseases. Negative contribution suggests that the gap in life expectancy would have been greater had it not been for lower mortality than in the 2019 North Atlantic. Therefore, the contribution of some causes or groups of causes can be greater than the total life expectancy gap.

© 2025 Karlsson O et al. *JAMA Network Open*.

eFigure 2. Contributions of Causes of Death to Life Expectancy Gaps Relative to the North Atlantic: Central & Eastern Europe and Latin America & the Caribbean, 2019

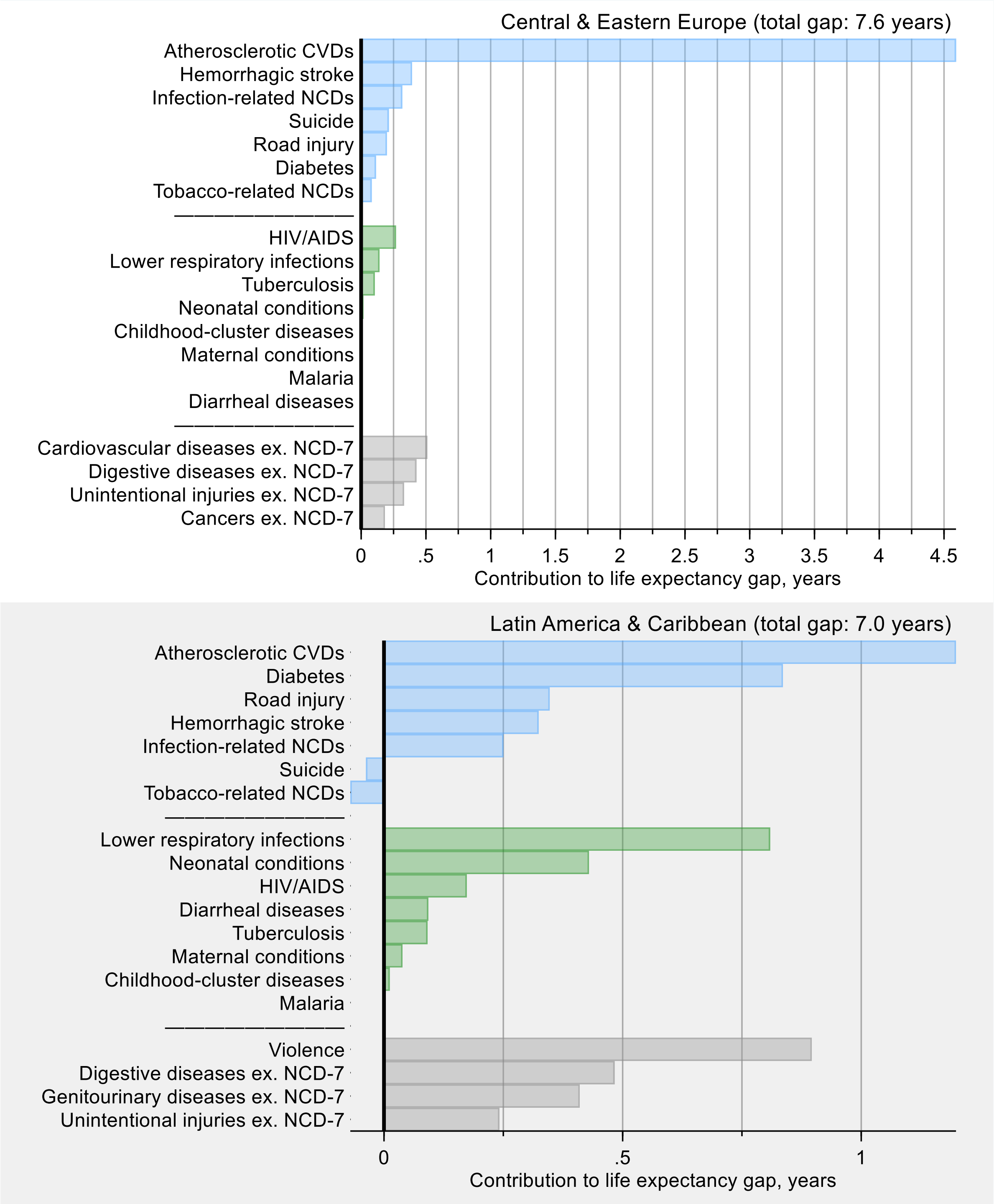

Note: Blue shows NCD-7, green I-8, and gray top 4 other causes (positive impact only). Comparisons were made to the 2019 North Atlantic (which had a life expectancy of 82 years). I-8 = 8 priority infectious and maternal and child health conditions. NCD-7 = 7 priority noncommunicable diseases (NCD) and injuries. ex. = excluding. CVD = cardiovascular diseases. Negative contribution suggests that the gap in life expectancy would have been greater had it not been for lower mortality than in the 2019 North Atlantic. Therefore, the contribution of some causes or groups of causes can be greater than the total life expectancy gap.

© 2025 Karlsson O et al. *JAMA Network Open*.

eFigure 3. Contributions of Causes of Death to Life Expectancy Gaps Relative to the North Atlantic: The Middle East & North Africa and the World, 2019

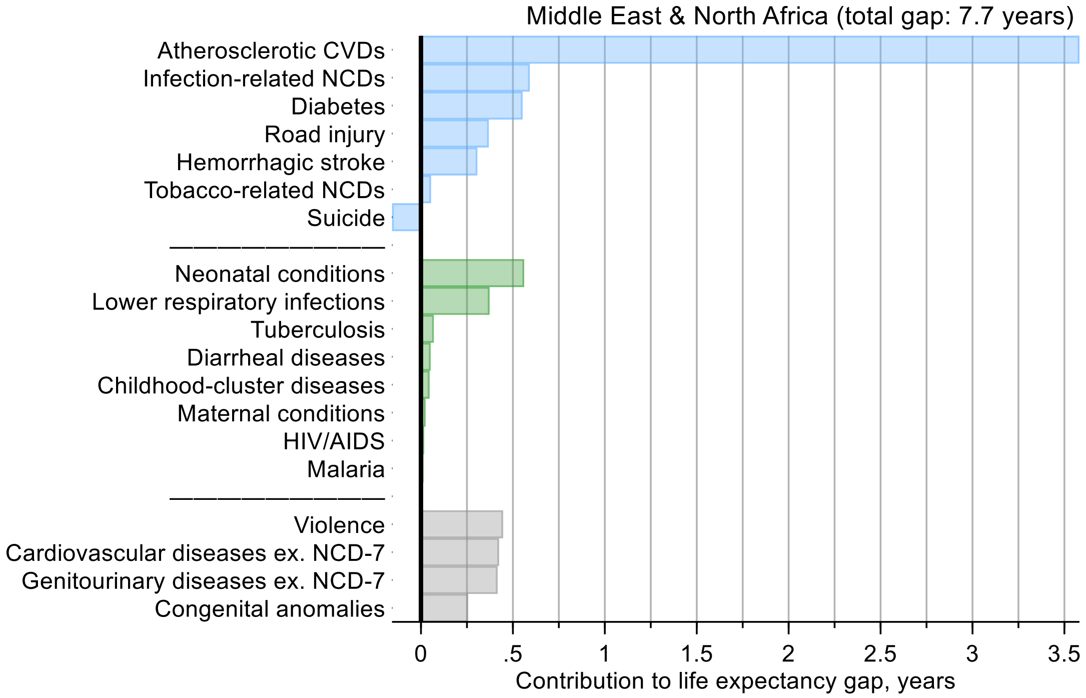

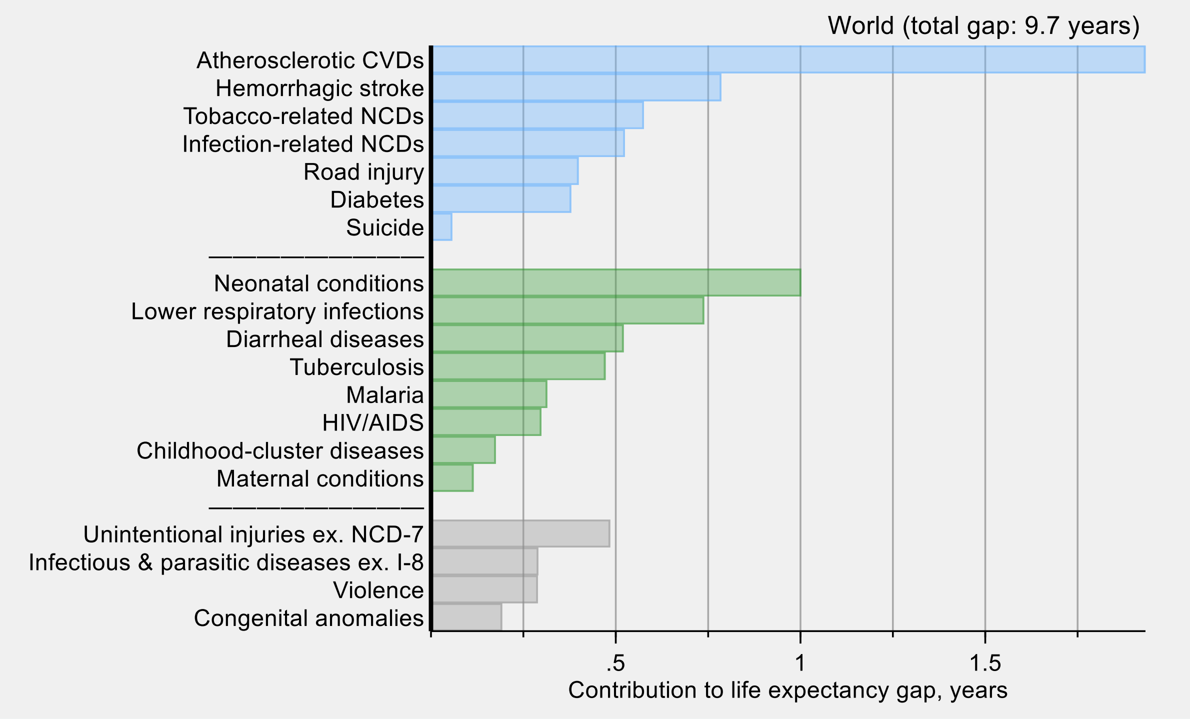

Note: Blue shows NCD-7, green I-8, and gray top 4 other causes (positive impact only). Comparisons were made to the 2019 North Atlantic (which had a life expectancy of 82 years). I-8 = 8 priority infectious and maternal and child health conditions. NCD-7 = 7 priority noncommunicable diseases (NCD) and injuries. ex. = excluding. CVD = cardiovascular diseases. Negative contribution suggests that the gap in life expectancy would have been greater had it not been for lower mortality than in the 2019 North Atlantic. Therefore, the contribution of some causes or groups of causes can be greater than the total life expectancy gap.

© 2025 Karlsson O et al. *JAMA Network Open*.

eTable 11. Tabulated Estimates from Figure 4: Percentage of Life Expectancy Gap Compared to the North Atlantic Attributable to Priority Conditions: Distribution Across Countries, 2019

	All			Low income			Middle income			High income		
	I-8	NCD-7	Both	I-8	NCD-7	Both	I-8	NCD-7	Both	I-8	NCD-7	Both
Minimum	-3.8	16	31	8.5	16	67	-1.9	18	48	-3.8	20	31
Percentile 5	1.4	20	59	22	17	68	5.1	22	62	-0.9	36	44
Percentile 25	11	30	71	42	22	72	12	36	75	6.0	49	64
Median	22	53	80	49	26	76	23	55	84	11	69	79
Percentile 75	42	73	88	54	30	79	36	72	89	18	82	90
Percentile 95	60	97	107	61	61	88	60	95	102	31	112	131
Maximum	67	150	160	66	77	99	67	150	160	35	115	137
Interquartile range	30	43	16	13	7.6	7.3	24	36	14	12	32	26
Mean	26	55	81	46	30	76	27	56	83	11	68	79
Standard deviation	19	26	17	13	14	6.7	18	25	16	9.0	23	25
Number of countries	165	165	165	26	26	26	103	103	103	32	32	32

Note: 'Both' shows I-8+NCD-7. Results are shown overall and by 2019 World Bank Income groups (four countries were not classified). Only countries with at least two years lower life expectancy than the 2019 North Atlantic (which had 82-year life expectancy) were included. Countries were equally weighted for descriptive statistics. Negative contribution and contribution over 100% suggest that the gap in life expectancy would have been greater had it not been for lower mortality for some causes than in the 2019 North Atlantic. I-8 = 8 priority infectious and maternal and child health conditions. NCD-7 = 7 priority noncommunicable diseases (NCD) and injuries. The I-8 are neonatal conditions, lower respiratory infections, diarrheal diseases, HIV/AIDS, tuberculosis, malaria, childhood-cluster diseases, and maternal conditions. The NCD-7 are atherosclerotic cardiovascular diseases (CVD), hemorrhagic stroke, NCDs strongly linked to infections, NCDs strongly linked to tobacco use, diabetes, road injury, and suicide.

© 2025 Karlsson O et al. *JAMA Network Open*.

eFigure 4. Country-Level Correlation Between the Percentage of Life Expectancy Gap Compared to the 2019 North Atlantic Attributable to the 15 Priority Conditions and the Total Life Expectancy Gap

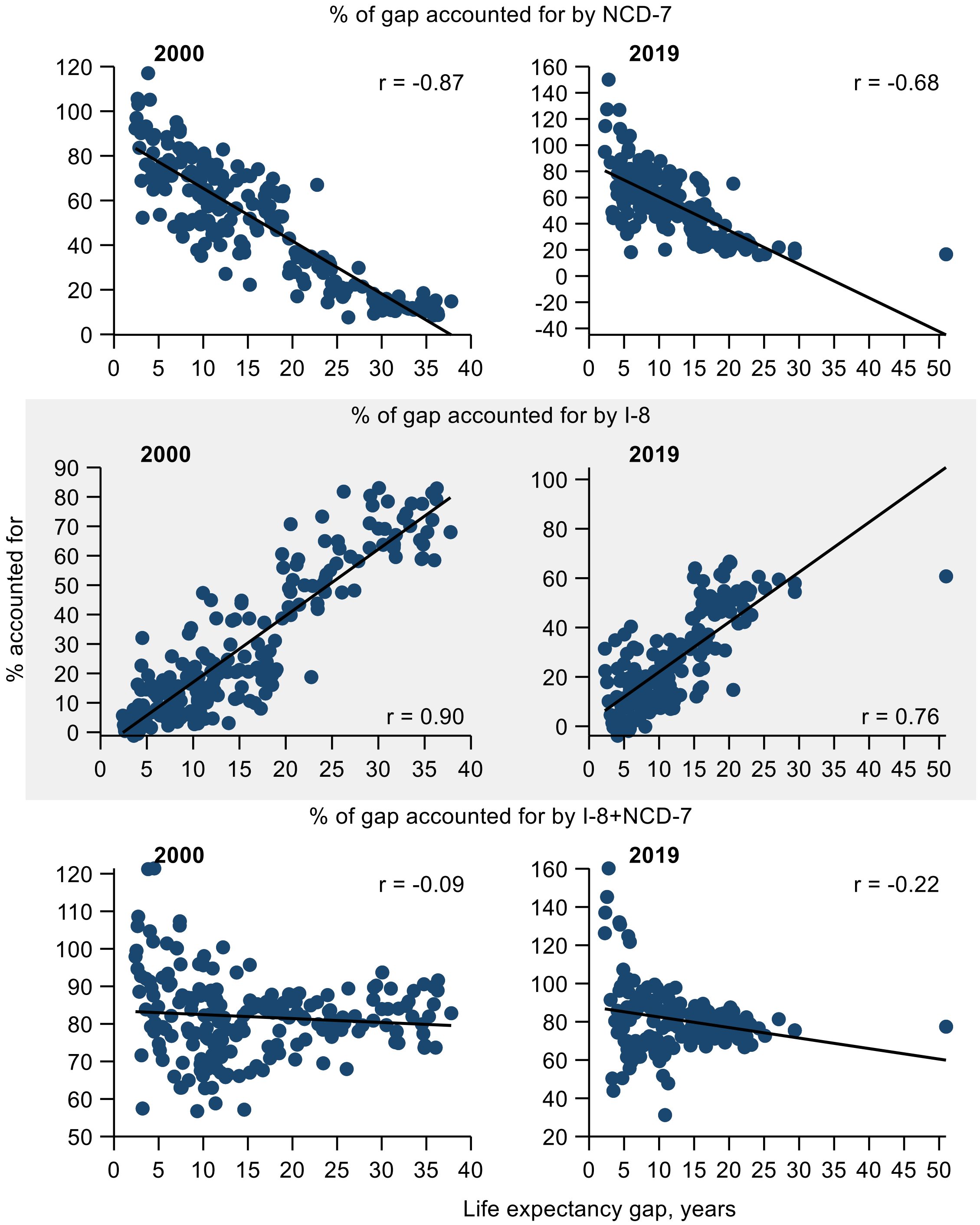

Note: Pearson's (r) correlation coefficients are shown. Countries were equally weighted. Both 2000 and 2019 were compared to the North Atlantic in 2019 (which had a life expectancy of 82 years). Only countries with at least two years lower life expectancy than the 2019 North Atlantic are included. Negative contribution and contribution over 100% suggest that the gap in life expectancy would have been greater had it not been for lower mortality for some causes than in the 2019 North Atlantic. I-8 = 8 priority infectious and maternal and child health conditions. NCD-7 = 7 priority noncommunicable diseases and injuries. The I-8 are neonatal conditions, lower respiratory infections, diarrheal diseases,

© 2025 Karlsson O et al. *JAMA Network Open*.

HIV/AIDS, tuberculosis, malaria, childhood-cluster diseases, and maternal conditions. The NCD-7 are atherosclerotic cardiovascular diseases (CVD), hemorrhagic stroke, noncommunicable diseases (NCD) strongly linked to infections, NCDs strongly linked to tobacco use, diabetes, road injury, and suicide.

© 2025 Karlsson O et al. *JAMA Network Open*.

eReferences

1. World Health Organization. *Global Health Estimates, 2000-2021*. Department of Data and Analytics (DNA) Division of Data, Analytics and Delivery for Impact (DDI) WHO; 2024.

2. World Health Organization. Global Health Estimates. Cause-specific mortality, 2000–2021. 2024. Accessed March 4, 2025. https://www.who.int/data/global-health-estimates

3. World Health Organization. *WHO Methods and Data Sources for Country-Level Causes of Death 2000-2021*. Department of Data and Analytics (DNA) Division of Data, Analytics and Delivery for Impact (DDI) WHO; 2024. Accessed March 4, 2025. https://cdn.who.int/media/docs/default-source/gho-documents/global-health-estimates/ghe2021_cod_methods.pdf?sfvrsn=dca346b7_1

4. World Population Prospects 2024, Online Edition. 2024. Accessed July 14, 2024. https://population.un.org/wpp/Download/Standard/MostUsed/

5. Pollard JH. On the decomposition of changes in expectation of life and differentials in life expectancy. *Demography*. 1988;25(2):265-276. doi:10.2307/2061293

6. Suchindran C, MEASURE Evaluation. Lesson 5: Expectation of Life at Birth. Multiple-Decrement Life Tables. 2024. Accessed July 14, 2024. https://www.measureevaluation.org/resources/training/online-courses-and-resources/non-certificate-courses-and-mini-tutorials/multiple-decrement-life-tables/lesson-5.html

7. Arriaga EE. Measuring and explaining the change in life expectancies. *Demography*. 1984;21(1):83-96. doi:10.2307/2061029

© 2025 Karlsson O et al. *JAMA Network Open*.
