# Supplementary material for: Priority Health Conditions and Global Life Expectancy Disparities
Source: JAMA Netw Open. 2025 May 23;8(5):e2512198. doi: 10.1001/jamanetworkopen.2025.12198 (PMC12102710; doi:10.1001/jamanetworkopen.2025.12198)
Supplement: Supplement 3. — Data Sharing Statement [file jamanetwopen-e2512198-s003.pdf]

Data Sharing Statement

Karlsson. Priority Health Conditions and Life Expectancy Disparities. *JAMA Netw Open*. Published May 23, 2025. doi:10.1001/jamanetworkopen.2025.12198

Data
Data available: Yes
Data types: Data (not involving human participants)
How to access data: Global Health Estimates are available from the World Health Organization upon request at healthstat@who.int. The UN WPP 2024 are available online at https://population.un.org/wpp/.
When available: With publication

Supporting Documents
Document types: Statistical/analytic code
How to access documents: All codes used in this paper is available at https://github.com/O-Karlsson/Priority-health-conditions-and-life-expectancy-disparities/.
When available: With publication

Additional Information
Who can access the data: Anyone
Types of analyses: Any
Mechanisms of data availability: Without investigator support
